# Mepolizumab improvements in health-related quality of life and disease symptoms in a patient population with very severe chronic rhinosinusitis with nasal polyps: psychometric and efficacy analyses from the SYNAPSE study

**DOI:** 10.1186/s41687-023-00543-5

**Published:** 2023-01-20

**Authors:** Wytske Fokkens, Andrew Trigg, Stella E. Lee, Robert H. Chan, Zuzana Diamant, Claire Hopkins, Peter Howarth, Valerie Lund, Bhabita Mayer, Ana R. Sousa, Steve Yancey, Maggie Tabberer, SYNAPSE study group, Ledit Ardusso, Miguel Bergna, María De Salvo, Pedro Elías, Gabriel García, Jorge Maspero, Ramón Rojas, Pablo Saez Scherbovsky, Alberto Tolcachier, Luis Wehbe, Anahí Yañez, Philip Bardin, Sara Barnes, Andrew Gillman, Richard Harvey, Chady Sader, Narinder Singh, Jaime Del Carpio, Marie-Noëlle Corriveau, Martin Desrosiers, Arif Janjua, Shaun Kilty, Doron Sommer, Leigh Sowerby, Peter Spafford, Christian Betz, Achim Beule, Adam Chaker, Mandy Cuevas, Moritz Groeger, Ludger Klimek, Heidi Olze, Carolina van Schaik, Martin Wagenmann, Barbara Wollenberg, Yury Yarin, Hyung-Ju Cho, Hun-Jong Dhong, Chang-Hoon Kim, Seontae Kim, Chae-Seo Rhee, Soo Whan Kim, Hyo Yeol Kim, Wytske J. Fokkens, Valeriu Bronescu, Corina Mella, Adriana Neagos, Doinel Radeanu, Catalin Stefan, Anton Edin, Sergey Karpischenko, Fatimat Khanova, Ekaterina Mirzabekyan, Andrey Ovchinnikov, Dmitriy Polyakov, Sergei Ryazantsev, Valeriy Svistushkin, Galina Tarasova, Vladimir Yakusevich, Cecilia Ahlström Emanuelsson, Johan Hellgren, Mattias Jangard, Anders Mårtensson, Karin Toll, Sean Carrie, Stephen Durham, Simon Gane, Jonathan Hobson, Claire Hopkins, Naveed Kara, Samuel Leong, Neil Massey, Guy Scaddin, Michael Armstrong, James Blotter, Matthew Brown, Timothy Courville, Cecelia Damask, Adam DeConde, Dale Ehmer, Adil Fatakia, Christine Franzese, Joseph Han, Thomas Higgins, Edward Kerwin, Craig LaForce, Stella Lee, Bradley Marple, Jonathan Matz, Chad McDuffie, Steven Miller, Jonathan Moss, Nayla Mumneh, Robert Nathan, Randall Ow, Jeffrey Rosenbloom, Rodney Schlosser, Heena Shah-Patel, Ronald Shealy, Ayesha Siddiqi, Stacey Silvers, Weily Soong, Richard Sterling, Neetu Talreja, Martha Tarpay, Luke Webb, H. James Wedner, Simon Wright, David Yen

**Affiliations:** 1 Department of Otolaryngology, University of Amsterdam, Amsterdam, The Netherlands; 2 Patient-Centred Outcomes, Adelphi Values, Bollington, Cheshire, UK; 3 Division of Otolaryngology, Brigham and Women’s Hospital, Harvard Medical School, Boston, MA USA; 4 Clinical Sciences, Respiratory, GSK, GSK House, Brentford, Middlesex, UK; 5 Department of Microbiology Immunology and Transplantation, KU Leuven, Catholic University of Leuven, Leuven, Belgium; 6 Department of Clinical Pharmacy and Pharmacology, University Medical Center Groningen, Groningen, The Netherlands; 7 Respiratory Medicine and Allergology, Skane University, Lund, Sweden; 8 ENT Department, Guys and St Thomas’s Hospital, King’s College, London, UK; 9 Global Medical Affairs, GSK, Brentford, Middlesex, UK; 10 Royal National Throat, Nose and Ear Hospital, University College London Hospitals, London, UK; 11 Clinical Statistics, GSK, GSK House, Brentford, Middlesex, UK; 12 Respiratory Therapeutic Area Unit, GSK, Research Triangle Park, NC USA; 13 Respiratory Patient Centered Outcomes, Value Evidence and Outcomes GSK, GSK House, Brentford, Middlesex, UK

**Keywords:** SNOT-22, VAS, Chronic rhinosinusitis with nasal polyps, Psychometric, Efficacy, Quality of life, Severity, Mepolizumab, Patient-reported outcome measures (PROMs)

## Abstract

**Background:**

Although the psychometric properties of patient-reported outcome measures (e.g. the 22-item Sino-nasal Outcomes Test [SNOT-22]) in chronic rhinosinusitis with nasal polyps (CRSwNP) have been defined, these definitions have not been extensively studied in patients with very severe CRSwNP, as defined by recurrent disease despite ≥ 1 previous surgery and a current need for further surgery. Therefore, the psychometric properties of the symptoms visual analogue scales (VAS) were evaluated, and meaningful within-patient change thresholds were calculated for VAS and SNOT-22.

**Methods:**

SYNAPSE (NCT03085797), a randomized, double-blind, placebo-controlled, 52-week trial, assessed the efficacy and safety of 4-weekly mepolizumab 100 mg subcutaneously added to standard of care in very severe CRSwNP. Enrolled patients (n = 407) completed symptom VAS (six items) daily and SNOT-22 every 4 weeks from baseline until Week 52. Blinded psychometric assessment of individual and composite VAS was performed post hoc, including anchor-based thresholds for meaningful within-patient changes for VAS and SNOT-22, supported by cumulative distribution function and probability density function plots. The effect of mepolizumab versus placebo for 52 weeks on VAS and SNOT-22 scores was then determined using these thresholds using unblinded data.

**Results:**

Internal consistency was acceptable for VAS and SNOT-22 scores (Cronbach’s α-coefficients ≥ 0.70). Test–retest reliability was demonstrated for all symptom VAS (Intra-Class Correlation coefficients > 0.75). Construct validity was acceptable between individual and composite VAS and SNOT-22 total score (r = 0.461–0.598) and between individual symptom VAS and corresponding SNOT-22 items (r = 0.560–0.780), based upon pre-specified ranges. Known-groups validity assessment demonstrated generally acceptable validity based on factors associated with respiratory health, with all VAS responsive to change. Mepolizumab treatment was associated with significantly increased odds of meeting or exceeding meaningful within-patient change thresholds, derived for this very severe cohort using six anchor groups for individual VAS (odds ratio [OR] 2.19–2.68) at Weeks 49–52, and SNOT-22 (OR 1.61–2.96) throughout the study.

**Conclusions:**

Symptoms VAS and SNOT-22 had acceptable psychometric properties for use in very severe CRSwNP. Mepolizumab provided meaningful within-patient improvements in symptom severity and health-related quality of life versus placebo, indicating mepolizumab provides substantial clinical benefits in very severe CRSwNP.

**Supplementary Information:**

The online version contains supplementary material available at 10.1186/s41687-023-00543-5.

## Plain English summary

Patients with chronic rhinosinusitis (CRS) often have blocked or runny noses, and loss of sense of smell. They can also have sac-like growths in their nose called nasal polyps, which often require surgical removement. The symptoms of CRS with nasal polyps can affect quality of life. In a clinical study named SYNAPSE, a new treatment option called mepolizumab reduced the size and severity of nasal polyps in patients suffering from very severe CRS with nasal polyps, compared with placebo. Mepolizumab also reduced the need for nasal polyp surgery. The SYNAPSE study also measured if 1 year of mepolizumab treatment improved patients’ symptoms and quality of life. This was evaluated by asking patients to complete two separate tasks. These tasks were rating symptoms on a visual analogue scale (VAS) and completing a quality of life questionnaire called SNOT-22. The objective of this analysis was to see if these questionnaires accurately assessed a patient’s quality of life. The analysis also assessed how many patients had major improvements in their symptoms with mepolizumab. Overall, data from 407 patients in the SYNAPSE study was analyzed. Results showed that both the VAS and SNOT-22 questionnaires accurately captured CRS symptoms and quality of life. In addition, patients treated with mepolizumab for 1 year had improvements in quality of life compared with placebo. In conclusion, these findings suggest that the VAS and SNOT-22 questionnaires are appropriate evaluation tools for patients with very severe CRS with nasal polyps. The findings also show that mepolizumab treatment is beneficial for these patients.

**Supplementary Information:**

The online version contains supplementary material available at 10.1186/s41687-023-00543-5.

## Background

Chronic rhinosinusitis with nasal polyps (CRSwNP) is a subtype of CRS, characterized by persistent inflammation of the paranasal sinuses [1–3]. Inflammation is typically eosinophilic in nature, driven by type 2 cytokines such as interleukin (IL)-4, IL-5, and IL-13 [4, 5], resulting in symptoms of nasal blockage, loss of sense of smell, nasal discharge and/or facial pain [1, 6]. The current standard of care (SoC) for CRSwNP includes intranasal corticosteroids, saline nasal irrigation, short courses of systemic corticosteroids (SCS) for exacerbations, and sinus surgery when appropriate medical therapy fails [1]. However, these treatments have substantial limitations, including adverse events associated with SCS use, and nasal polyp (NP) recurrence following surgery [7, 8], highlighting the need for new, effective treatment options.

The anti-IL-5 humanized monoclonal antibody mepolizumab is approved for the treatment of CRSwNP in the US and EU [9–11]. In patients with CRSwNP in previous Phase II trials, mepolizumab has been shown to improve symptoms, reduce NP size and reduce the need for sinus surgery compared with placebo [12, 13]. In addition, results from SYNAPSE, a Phase III trial have shown that in adults with very severe CRSwNP in need of revision surgery, 4-weekly mepolizumab 100 mg subcutaneously (SC) plus SoC significantly improved NP size and nasal obstruction versus placebo, with no new safety findings found in addition to those previously reported in patients with CRSwNP treated with mepolizumab [12–14].

Since CRSwNP significantly impacts health-related quality of life (HRQoL) [12, 15, 16], clinical trials assessing the efficacy of novel treatments in patients with CRSwNP have included patient-reported outcome measures (PROMs) to evaluate symptom severity (HRQoL via the use of, for example, the visual analogue scale (VAS) and Sino-nasal Outcome Test (SNOT)-22, respectively, as supported by the European Position Paper on Rhinosinusitis and Nasal Polyposis (EPOS 2020) [1]. The psychometric properties of the VAS were assessed in a Phase II study assessing mepolizumab clinical efficacy in CRSwNP [17]. The analyses indicated that although overall patient comprehension of the VAS was good, there was room for improvement. Consequently, to improve clarity (and therefore data quality) of the VAS, modifications were made to the item names and the verbal descriptors, the facial pain/pressure VAS item was added, and a 24-h recall period was implemented for use in the Phase III SYNAPSE study [14, 18]. As VAS is a key endpoint in CRSwNP research, it was necessary to repeat the psychometric analysis of the modified VAS using Phase III data. Furthermore, as the score interpretation thresholds available to assess treatment response in CRSwNP trials are based on a CRSwNP population of mixed severity [19, 20], information on thresholds required for patients with the most severe disease was needed as the burden of symptoms in patients with severe CRSwNP is high and has a significant impact on HRQoL [21]. Indeed, the need for different thresholds for patients with the most severe disease has been demonstrated in the asthma field [19, 22]. As such, the objectives of this post hoc analysis were two-fold. First, to evaluate the psychometric properties of VAS scores and derive meaningful within-patient change thresholds for VAS and SNOT-22 in patients with very severe CRSwNP using blinded data from SYNAPSE. Second, patients were classified as responders or non-responders using meaningful within-patient change thresholds thereby assessing the efficacy of mepolizumab 100 mg SC administered every 4 weeks on symptom severity and HRQoL in adults with very severe CRSwNP.

## Methods

### Study design and patients

SYNAPSE was a Phase III randomized, double-blind, placebo-controlled, parallel-group trial (GSK205687; NCT03085797; Additional file 1: Figure S1) [14]. Patients (N = 407) were randomized (1:1) to mepolizumab 100 mg SC (n = 206) or placebo (n = 201) every 4 weeks, for 52 weeks in addition to SoC, including daily mometasone furoate nasal spray throughout the study period, saline nasal irrigations, and courses of SCS and/or antibiotics, as required [14].

Patient eligibility criteria included having a diagnosis of very severe CRS, characterized by (1) the presence of recurrent, refractory severe bilateral NP symptoms, (2) ≥ 1 prior surgery for NPs (a procedure involving instruments with resulting tissue removal) in the past 10 years, and 3) a current need for surgery (defined as an overall symptoms VAS score > 7 and endoscopic NP score of ≥ 5 [maximum 8], with a score ≥ 2 in each nasal cavity). Inclusion criteria for this study resulted in patients with very severe CRSwNP [14].

### VAS and SNOT-22 assessments

Patients completed six individual VAS assessments daily (nasal obstruction, nasal discharge, mucus in the throat, loss of sense of smell, facial pain, and overall symptoms) using an electronic diary (eDiary) and a recall period of 24 h. For each VAS, patients rated their symptom on a scale from ‘none’ (0) to ‘as bad as you can imagine’ (100). Results were transformed linearly to a 0–10 range for reporting (higher scores indicated greater symptom severity); the psychometric properties were unaffected by this transformation.

Patients also completed SNOT-22 assessments every 4 weeks using the same electronic device and a recall period of 2 weeks. A total of 22 symptoms were rated on a 0–5 scale, with a final total score of 0–110 (higher scores indicated greater disease impact on HRQoL). Endpoints from SYNAPSE relevant to this analysis are listed in Additional file 1: Supplementary Methods.

### Psychometric assessments

Blinded assessments of VAS and SNOT-22 were performed post hoc using data from the intent-to-treat population. Each VAS item was assessed separately, in addition to proposed four-item composite (nasal symptoms) and five-item composite (nasal symptoms and facial pain) scores (Additional file 1:Figure S2). Daily VAS scores at the Week 20 visit were used for confirmatory factor analysis (CFA) and internal consistency analyses; 4-week average VAS scores were used for analyses of the VAS over time, for example, test–retest (assessed at Week 20 and Week 24). Key analysis time points for efficacy assessments were Baseline, Weeks 20, 24, and 52. Where available, Week 20 data were prioritized for cross-sectional psychometric analyses as this time point was expected to have maximal change from baseline in individual patient scores while maximizing the number of patients included in the analyses (minimal patient drop out at this time point). Floor and ceiling effects, test–retest reliability, known-group validity, and ability to detect change were not assessed for SNOT-22 as these have been previously demonstrated [14]. No imputation of missing data was performed; 4-week averages were derived based on all available data within that timeframe.

#### Item characteristics

VAS ceiling and floor effects were explored, defined as > 15% of patients selecting the most severe health state (‘worst imaginable’ [ceiling]) or least severe health state (‘none’ [floor]) [23]. However, owing to the severity of the CRSwNP in patients included in this study, it was anticipated that scores would be distributed towards the higher end of the scale at baseline and ceiling effects may have been present at this time point. Inter-item correlations were therefore assessed for the individual symptom VAS scores (Spearman’s correlations to allow for violation of normality) and the SNOT-22 items (polychoric) at Week 20. Items that correlated very highly with one another (r ≥ 0.90) were flagged as potentially indicating redundancy.

#### Domain structure

CFA was performed to summarize the shared variance between items for the nasal symptoms composite VAS score, nasal symptoms and facial pain composite VAS score and SNOT-22 hypothesized domain structure [24, 25] using Week 20 data. Maximum likelihood estimator was used for continuous VAS items. A standardized loading of > 0.40 was considered indicative of an item as an adequate indicator of the factor [26]. Global model fit was assessed using Chi-square (values *P* ≥ 0.05 indicate acceptable fit), comparative fit index (CFI; values ≥ 0.95 indicate acceptable fit), standardized root mean residual (SRMR; values < 0.10 are considered acceptable), and root mean square error of approximation (RMSEA) models (values < 0.10 are considered acceptable) [24, 27, 28].

#### Reliability

Internal consistency, the extent to which responses to individual items within a score are interrelated, was investigated for both VAS composite scores and the SNOT-22 by calculating Cronbach’s α coefficient for each score, with a coefficient ≥ 0.70 considered acceptable [29]. Test–retest reliability, the degree to which scores are similar between two time points in patients who are stable, was evaluated for the individual symptoms VAS, overall symptoms VAS and both VAS composite scores by calculating the Intra-Class Correlation (ICC) coefficient for VAS and assessing stability between Weeks 20 and 24 (Additional file 1: Table S1). The ICC was based on a multiple measurement, absolute agreement, two-way random effects model (equivalent to ICC 2,k) [30]. Samples of all stable patients (between Weeks 20 and 24) were defined as patients that had no improvement or worsening according to other measures (detailed in Additional file 1: Supplementary Methods). Test–retest reliability was interpreted as follows: ICC < 0.5 poor reliability, 0.75 > ICC ≥ 0.5 moderate reliability, ICC ≥ 0.75–0.9 good reliability, and ICC > 0.90 excellent reliability [30] (See Additional file 1: Supplementary Methods for the formula).

#### Validity

Construct validity, whether similar concepts are more correlated than dissimilar concepts, was assessed at Week 20 for individual and overall symptoms VAS and both composite VAS scores using Spearman’s correlations. A priori hypothesized construct validity correlations were assessed with the following measures: The University of Pennsylvania Smell Identification Test (UPSIT) [31], endoscopic NP score [32], Peak Nasal Inspiratory Flow (PNIF) [33], SNOT-22 total score and individual items [34, 35], 36-item Short Form Health Survey (SF-36v2; Physical and Mental Component Summary scores [PCS and MCS]) [36], and Work Productivity and Activity Impairment (WPAI) Questionnaire (absenteeism, presenteeism, work productivity loss and activity impairment) (Additional file 1:Table S2) [37]. Correlations were based on predefined ranges (high: ≥ 0.50, medium: ≥ 0.30 to  < 0.50, low: < 0.30) [38].

The known-groups method (to differentiate between clinically distinct groups [29]) was used to evaluate the construct validity of the individual and overall symptoms VAS and both composite VAS scores. VAS scores were compared in patients grouped according to ranges of key baseline characteristics: comorbid asthma, baseline blood eosinophil count categories, non-steroidal anti-inflammatory drug-exacerbated respiratory disease (N-ERD), Asthma Control Questionnaire (ACQ)-5 score in patients with comorbid asthma and number of prior surgeries [39]. Between-group effect size estimates were calculated as per Hedges 1981 [39], and effect sizes (ES) were interpreted as small (ES = 0.20), moderate (ES = 0.50), and large (ES = 0.80) [38]. The statistical significance (*P* ≤ 0.05) of differences in scores between groups was calculated using the F-test of one-way analysis of variances.

#### Responsiveness

Ability to detect change was determined by assessing change between baseline to Week 52 in individual and overall symptoms VAS and both composite VAS scores in patients believed to have experienced change (improvement or worsening) versus patients understood to be stable. Improved, stable and worsened scores, respectively, were defined as ≤ − 1, 0, ≥ 1-point changes for endoscopic NP score, ≥ 20, < − 20– < 20, ≤ − 20 L/min changes for PNIF, ≤  − 2, > − 2– < 2, ≥ 2-point changes for overall VAS symptom score, ≤ − 8.9, − 8.9– < 8.9, ≥ 8.9-point changes for SNOT-22 total score, and ≤ − 2, − 2– < 2, ≥ 2-point changes for SNOT-22 domain scores (nasal obstruction, loss of taste or smell, thick nasal discharge, facial pain/pressure, post-nasal discharge). Mean change scores were compared within and between groups and interpreted as described above for known-groups validity assessments.

#### Interpretation of scores: meaningful within-patient improvements

Thresholds for meaningful within-patient change were derived to determine the proportion of responders for subsequent unblinded analyses. Anchor-based analyses were performed for individual and overall VAS symptoms and both composite VAS scores and the SNOT-22 total score using data from baseline to Week 52. Potential anchors included in this exploratory analysis were endoscopic NP score, PNIF, overall VAS and SNOT-22, none of which have been established as verified anchors and no meaningful changes have been reported for overall VAS and endoscopic NP scores. Polyserial correlations were used to assess the relationship between potential anchors and change in VAS scores; anchors with a coefficient of ≥ 0.3 were selected [40]. Anchors that were deemed to have a sufficient relationship with the PROM scores were used to define patients as minimally improved or stable as described in Additional file 1: Table S3. Definition justifications are also provided in Additional file 1: Table S3 and were based on clinical insight, published literature and patient input. As recommended by the Food and Drug Administration, [41, 42] descriptive statistics within each anchor category were supplemented with cumulative distribution function (CDF) and probability density function (PDF) plots. These were split by anchor categories to compare estimated thresholds for meaningful within-patient changes in VAS scores and SNOT-22 scores from baseline to Week 52 in this very severe CRSwNP population. The use of group-level statistics to estimate within-individual change thresholds has been contested owing to individual patients varying in their own personal threshold for meaningful improvement; however, when the objective of an analysis is to use these thresholds to estimate the proportion of responders in a population, this estimated responder rate should still be valid [43]. In addition, it has been recommended that the statistical significance of individual-level change should inform use of within-patient thresholds; [44] this was assessed by the 95% coefficient of repeatability (1.96*√2*standard error of measurement).

#### Response to treatment

Mepolizumab efficacy versus placebo on VAS and SNOT-22 was determined using the meaningful within-patient change thresholds for individual and overall symptoms VAS and for the SNOT-22 total score. Meaningful within-patient changes were analyzed using a logistic regression model with covariates of treatment group, geographic region, baseline score, and log(e) baseline blood eosinophil count. Additional analysis details are included in Additional file 1: Supplementary Methods.

## Results

### Patient population

Of the 407 patients included in SYNAPSE, 206 received mepolizumab and 201 received placebo. Overall, patients were mostly male (65%) with a mean (SD) age of 48.8 (13.0) years. The median baseline VAS and SNOT-22 total scores were similar between treatment groups (Table 1).

**Table 1:** Baseline demographics and clinical characteristics

	Placebo (n = 201)	Mepolizumab 100 mg SC (n = 206)	Total (n = 407)
Age, years, mean (SD)	48.9 (12.5)	48.6 (13.6)	48.8 (13.0)
Male, n (%)	125 (62)	139 (67)	264 (65)
Blood eosinophil count, cells/µL, geometric mean (standard logs)	400 (0.775)	390 (0.755)	–
Patients with asthma, n (%)	149 (74)	140 (68)	289 (71)
Patients with N-ERD, n (%)	63 (31)	45 (22)	108 (27)
Nasal surgeries in previous 10 years			
1	81 (40)	108 (52)	189 (46)
2	47 (23)	47 (23)	94 (23)
> 2	73 (36)	51 (25)	124 (30)
Duration of NP, years, mean (SD)	11.46 (8.27)	11.36 (8.52)	11.41 (8.39)
VAS score*, median (range)			
Nasal obstruction	9.1 (5.3–10.0)	9.0 (6.5–10.0)	9.1 (5.3–10.0)
Loss of sense of smell	10.0 (6.7–10.0)	10.0 (0.9–10.0)	10.0 (0.9–10.0)
Overall symptoms	9.2 (7.2–10.0)	9.1 (7.2–10.0)	9.2 (7.2–10.0)
Nasal discharge	9.0 (1.4–10.0)	8.9 (1.0–10.0)	–
Mucus in throat	9.1 (0.5–10.0)	8.9 (0.2–10.0)	–
Facial pain	8.9 (0.0–10.0)	8.5 (0.0–10.0)	–
SNOT-22 total score, median (range)*	64.0 (19–110)	64.0 (17–105)	64.0 (17–110)
Total endoscopic score, median (range) (scale: 0–8)	6.0 (0–8)	5.0 (2–8)	–
PNIF, median (range)	90.0 (0–380)	92.5 (0–350)	–
ACQ-5 score, mean (SD)^†^	2.2 (1.4)	2.4 (1.4)	–

*ACQ-5* Asthma Control Questionnaire-5, *N-ERD* Non-steroidal anti-inflammatory drug exacerbated respiratory disease, *NP* Nasal polyp, *PNIF* peak nasal inspiratory flow, *SC* Subcutaneous, *SD* Standard deviation, *SNOT-22* Sino-nasal outcome test-22, *VAS* Visual analogue scale

*Higher scores indicate greater disease severity or worse health-related quality of life

^†^In patients with asthma only, placebo n = 144, mepolizumab n = 138

### VAS psychometric analysis

#### Item characteristics

The individual symptom VAS scores were clustered at the high end of the scale at baseline, owing to the severity of symptoms present in the population. VAS responses were distributed across the full response scale at Weeks 20, 24 and 52. Ceiling effects were present at all time points for the loss of smell VAS, and a floor effect was observed for the facial pain or pressure VAS at Week 52. While no other VAS demonstrated substantial floor or ceiling effects, a higher proportion of responses were clustered at the upper versus lower end of the scale for all VAS scores at baseline (as was expected owing to the eligibility criteria), but not during the treatment period (Additional file 1:Figure S3).

All correlations between single VAS items were acceptable (> 0.3), with correlations > 0.9 observed only between two pairs of VAS: nasal obstruction VAS and nasal discharge VAS (r = 0.929), and nasal discharge VAS and mucus in throat VAS (r = 0.900). While a correlation of > 0.9 may show proof of redundancy, all items were included in the final model, as these item pairs include symptoms highly important to patients [21].

#### Domain structure

CFA using Week 20 data found that standardized loadings were ≥ 0.40 for the unidimensional nasal symptoms composite VAS score, and nasal symptoms and facial pain composite VAS score. Further details of model fit are included in Additional file 1: Supplementary Results. Residual correlations were not suggested (by modification indices > 10, to capture a critical value of 10.83 corresponding to *P* < 0.001 [45]) between the pairs of items with high inter-item correlations but were suggested between nasal obstruction and loss of smell.

#### Reliability

Cronbach’s α coefficients indicated acceptable internal consistency and reliability. For both nasal symptoms composite VAS score, and nasal symptoms and facial pain composite VAS scores, coefficients exceeded the predefined acceptable threshold of ≥ 0.70 at Week 20 (0.910 and 0.926) and Week 52 (0.904 and 0.926). To evaluate test–retest reliability, ICC coefficients between scores at Weeks 20 and 24 were assessed in a subset of participants defined as stable (Additional file 1: Table S1). All ICCs for individual symptom VAS, Overall VAS and both composite scores exceeded the prespecified threshold of > 0.75, with the lower 95% confidence intervals (CIs) of all estimates > 0.90 (indicating ‘excellent’ reliability).

#### Validity

Construct validity was acceptable between individual and composite VAS and the SNOT-22 total score (r = 0.461–0.598), and between individual symptom VAS and corresponding SNOT-22 items (r = 0.560–0.780), exceeding a priori hypothesized correlations (Additional file 1: Table S2). A priori hypothesized correlations between loss of smell VAS and UPSIT (r = − 0.494), and other non-loss of smell VAS scores and UPSIT (r = 0.211 to − 0.239), endoscopic NP (r = 0.199–0.279) and PNIF (r = − 0.216 to − 0.243) were not met. Hypothesized weak associations between VAS scores and WPAI work missed (r = 0.129–0.167) and SF-36 MCS (r = − 0.149 to − 0.216) were met, but associations between VAS scores and WPAI impairment-based scores (r = 0.370–0.553) and SF-36 PCS (r = − 0.308 to − 0.367; except loss of smell VAS) exceeded predictions (Additional file 1: Table S2).

Known-groups validity assessment demonstrated generally acceptable validity based on comorbid asthma, blood eosinophil count and N-ERD. Statistically significant between-group differences (F-test *P* < 0.05), with small to large effect sizes (ES range 0.20 to 0.80), on several VAS were noted for ACQ-5 score and number of prior surgeries at Week 20 (Additional file 1: Table S4).

#### Responsiveness

In the ability to detect change analysis, effect sizes (− 3.61 to − 8.84) reported using all anchors indicated very large changes in the improved groups across all VAS and both composite scores. Changes in VAS scores were also large within the stable groups (ES − 0.48 to − 5.02), although always smaller than those in the improved groups.

#### Interpretation of scores

Anchors based on the SNOT-22 (polyserial correlation coefficient range: − 0.436 to − 0.599) and overall symptoms VAS (polyserial correlation coefficient range: − 0.768 to − 0.974) were sufficiently correlated (r ≥ 0.3) with change in VAS scores and thus suitable for use. Mean anchor-based changes from baseline to Week 52 in VAS scores for patients categorized as stable or minimally improved (Table 2), CDF plots (Additional file 1: Figures S4–S10) and PDF plots informed meaningful within-patient change improvement thresholds of − 2.5 points for the overall symptoms, nasal discharge, and facial pain VAS, and − 3.0 points for the nasal obstruction, loss of sense of smell, and mucus in throat VAS. The absolute magnitude of these thresholds (threshold divided by SD) ranged from 0.76 to 1.14 in standardized units. The selection of these thresholds was largely driven by the CDF plots, where each was generally deemed sensitive enough to capture most minimally improved patients while specific enough to exclude the majority of stable patients across the different anchors. Estimates for the 95% coefficient of repeatability ranged from 1.00 to 2.13, suggesting that all thresholds represent statistically significant within-individual changes [44].

**Table 2:** Anchor-based change from baseline to Week 52 for individual VAS scores

Anchor	Nasal obstruction	Loss of sense of smell	Overall symptom	Nasal discharge	Mucus in throat	Facial pain
	Mean (95% CIs)					
Overall VAS score*						
Minimal improvement n = 49	− 3.16 (− 3.42, − 2.90)	− 1.11 (− 1.58, − 0.65)	–	− 3.25 (− 3.65, − 2.86)	− 3.25 (− 3.77, − 2.73)	− 2.99 (− 3.46, − 2.52)
Stable n = 85	− 0.45 (− 0.63, − 0.26)	− 0.33 (− 0.50, − 0.16)		− 0.55 (− 0.87, − 0.23)	− 0.54 (− 0.91, − 0.16)	− 0.49 (− 0.88, − 0.11)
SNOT-22 total score^†^						
Minimal improvement, n = 31	− 2.80 (− 3.70, − 1.90)	− 1.51 (− 2.46, − 0.57)	− 2.94 (− 3.92, − 1.97)	− 2.58 (− 3.51, − 1.65)	− 2.33 (− 3.37, − 1.29)	− 2.62 (− 3.64, − 1.59)
Stable, n = 38	− 1.77 (− 2.61, − 0.93)	− 1.05 (− 1.83, − 0.27)	− 1.84 (− 2.69, − 0.99)	− 1.80 (− 2.64, − 0.96)	− 2.07 (− 2.94, − 1.20)	− 1.58 (− 2.50, − 0.66)
*SNOT-22 nasal obstruction*^*‡*^						
Minimal improvement, n = 86	− 4.66, (− 5.23, − 4.09)	–	–	–	–	–
Stable, n = 113	− 2.62 (− 3.12, − 2.13)					
*SNOT-22 loss of taste or smell*^*‡*^						
Minimal improvement, n = 43	−	− 4.35 (− 5.24, − 3.46)	–	–	–	–
Stable, n = 187		− 1.05 (− 1.34, − 0.75)				
*SNOT-22 thick nasal discharge*^*‡*^						
Minimal improvement, n = 81	–	–	–	− 5.08 (− 5.76, − 4.39)	–	–
Stable, n = 113				− 2.75 (− 3.27, − 2.23)		
*SNOT-22 post-nasal discharge*^*‡*^						
Minimal improvement, n = 75	–	–	–	–	− 4.83 (− 5.49, − 4.17)	–
Stable, n = 137					− 2.96 (− 3.47, − 2.44)	
*SNOT-22 facial pain/pressure*^*‡*^						
Minimal improvement, n = 78	–	–	–	–	–	− 4.82 (− 5.51, − 4.14)
Stable, n = 130						− 2.66 (− 3.18, − 2.13)

*CI*, confidence interval, *SNOT-22* Sino-nasal outcome test-22, *VAS* Visual analogue scale

*Minimal improvement − 2 ≥ change score > − 4, stable -2 < change score < 2

^†^Minimal improvement − 8.9 ≥ change score > − 17.8, stable − 8.9 < change score < 8.9

^‡^Minimal improvement − 2, stable − 2 < change score < 2

### SNOT-22 psychometric analysis

#### Item characteristics

Inter-item correlations between each possible pairing of the 22 individual items of the SNOT-22 were medium to high (r > 0.3) and there was no item redundancy (i.e. r ≥ 0.9).

#### Reliability and validity

**Fig. 1:**
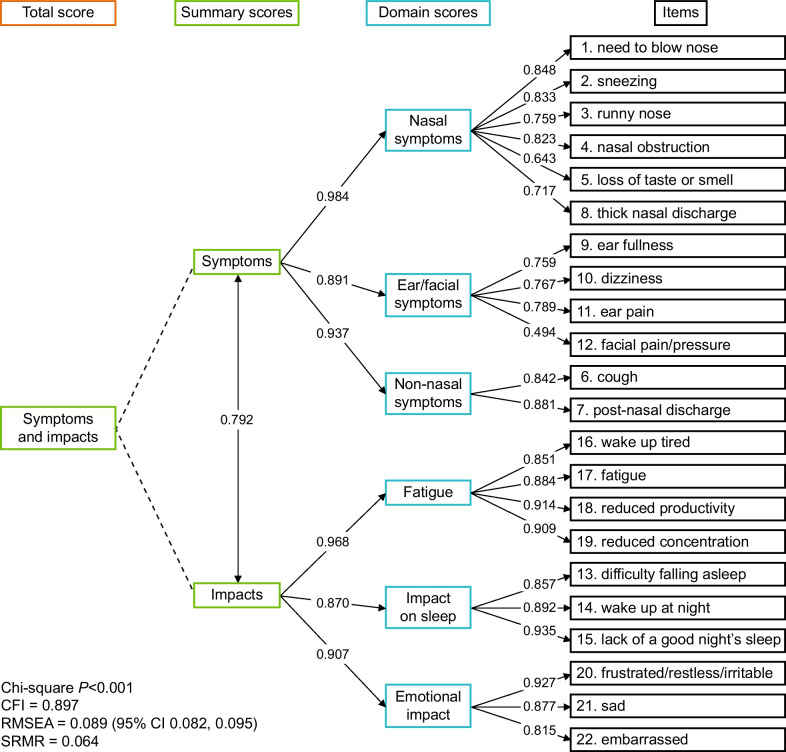
The six-domain SNOT-22 model with standardized factor loadings. The CFA model, including the standardized factor loadings (numbers on arrows) from each hypothesized domain to the SNOT-22 items and Chi-squared, CFI, RMSEA, and SRMR fit statistics. Standardized factor loadings represent the correlation coefficients between factors. *CFA* Confirmatory factor analysis, *CFI* Comparative fit index, *RMSEA* Root mean square error of approximation, *SNOT-22* Sino-nasal outcome test-22, *SRMR* Standardized root mean square residual

Cronbach’s α was ≥ 0.70 at Weeks 20 (0.963) and 52 (0.961), further supporting acceptable internal consistency reliability of the SNOT-22 total score. The CFA of SNOT-22 data supported the six-domain model with second order symptoms and impact factors (Fig. 1). All standardized loadings were ≥ 0.40, indicating that all SNOT-22 items were adequate indicators of their respective factors [26], and the validity of the SNOT-22 total score was also supported due to the high correlation between the second order symptoms and impact factors (r = 0.792). Global fit statistics were acceptable for RMSEA (0.089) and SRMR (0.064) but not CFI (0.897) and Chi-square (*P* < 0.001).

#### Interpretation of scores

**Fig. 2:**
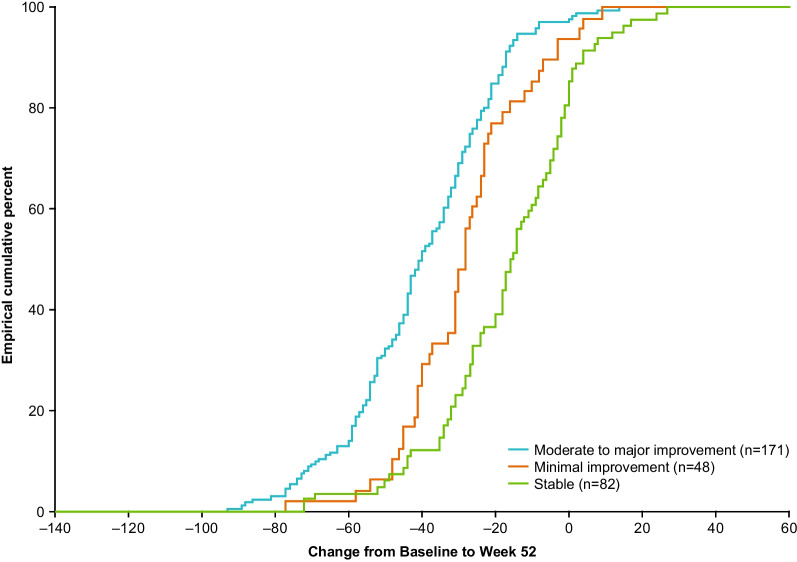
Mean change in SNOT-22 score by VAS anchor in total ITT population. Overall VAS anchor groups were defined by change in overall VAS symptom score from baseline to Week 52: moderate to major improvement: ≤  − 4-point change in VAS score; minimal meaningful improvement: ≤ − 2 to > − 4-point change in VAS score; limited improvement or worsening (stable): > − 2 to < 2− point change in VAS score; *CDF* Cumulative distribution function, *CI* Confidence interval, *ITT* Intent-to-treat, *SNOT-22* Sino-nasal outcome test-22, *VAS* Visual analogue scale

**Fig. 3:**
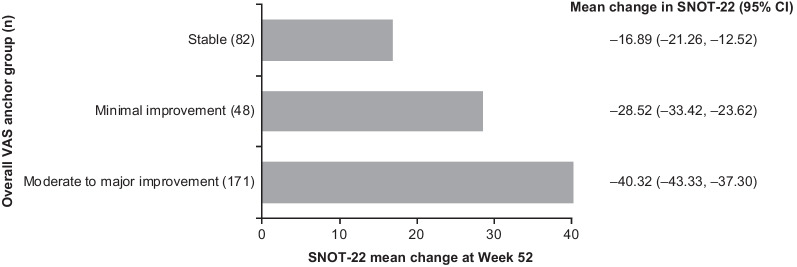
CDF plot: change from baseline in SNOT-22 total score by overall VAS anchor. Overall VAS anchor groups were defined by change in overall VAS symptom score from baseline to Week 52: moderate to major improvement: ≤ − 4-point change in VAS score; minimal meaningful improvement: ≤ − 2 to > − 4-point change in VAS score; limited improvement or worsening (stable): > − 2 to < 2-point change in VAS score. *CDF* Cumulative distribution function, *SNOT-22* Sino-nasal outcome test-22, *VAS* Visual analogue scale

Although overall symptoms VAS is not a verified anchor, it was identified as the overall measure of stability and improvement in patients with severe CRSwNP, as this was the only anchor sufficiently correlated (r ≥ 0.3) with SNOT-22 scores (polyserial correlation coefficient: − 0.558). Using the overall symptoms VAS as an anchor to classify patients as stable or minimally improved (minimal improvement was arbitrarily defined as − 2 ≥ change score ≥ − 4, based on EPOS guidance for severity [1]), the mean (95% CI) within-patient change in SNOT-22 total score (analyzed using observed data from 301 patients) for patients reporting minimal improvement was − 28.52 (− 33.42, − 23.62) points (Fig. 2). CDF (Fig. 3) and PDF plots suggested that a − 28-point change was sensitive enough to capture most improved patients while being specific enough to exclude the majority of stable patients, within this very severe population. The absolute magnitude of this threshold (threshold divided by SD) was 1.25 in standardized units. This 28-point threshold is close to 50% of the mean change from baseline in SNOT-22 total score observed in SYNAPSE. Therefore, 50% of the baseline score may be a plausible general threshold to use beyond this particular, very severe CRSwNP population. The 95% coefficient of repeatability was 11.89, suggesting a threshold of ≥ 28 points represents a statistically significant within-patient change [44].

### VAS response to treatment

**Fig. 4:**
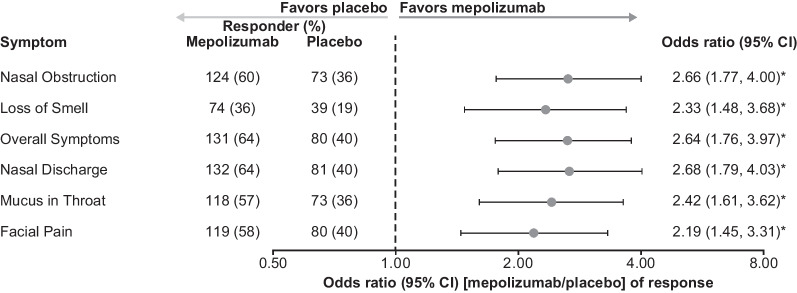
Probability of meaningful within-patient change^†^ in VAS score (Weeks 49–52). *P < 0.001; ^†^ ≥ 2.5-point change (improvement) for overall symptoms, nasal discharge, and facial pain VAS scores, and a ≥ 3-point change (improvement) for nasal obstruction, loss of sense of smell, and mucus in throat VAS scores. *CI* Confidence interval, *VAS* Visual analogue scale

**Fig. 5:**
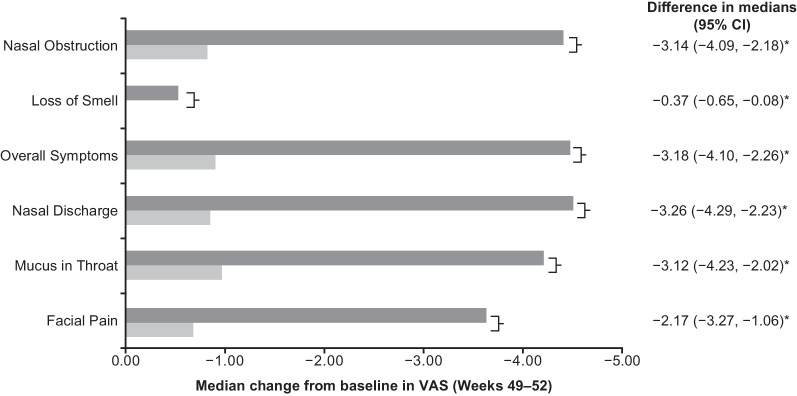
Change from baseline at Weeks 49–52 in individual VAS scores. *P < 0.001. *CI* Confidence interval, *VAS* Visual analogue scale

**Fig. 6:**
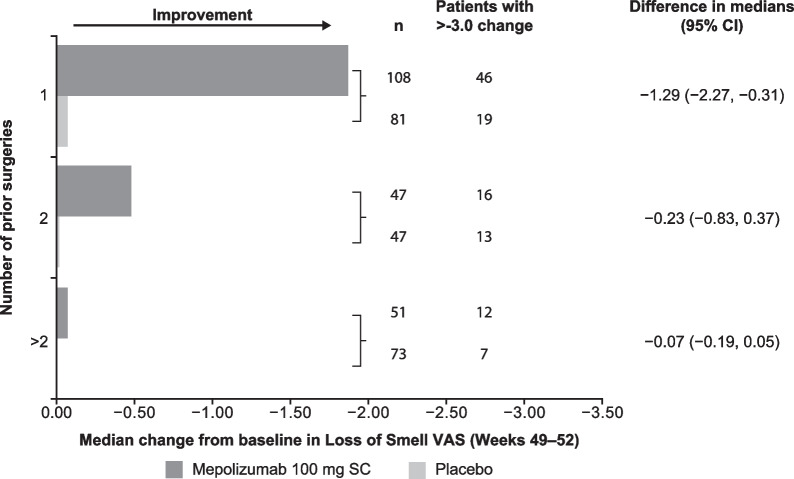
Change from baseline in loss of sense of smell VAS score by surgery number. *CI* Confidence interval, *SC* Subcutaneous, *VAS* visual analogue scale

Compared with placebo, patients receiving mepolizumab had significantly increased odds (odds ratio [OR] 2.19–2.68) of achieving a meaningful improvement in individual VAS scores, based on meeting or exceeding the meaningful within-patient change thresholds at Weeks 49–52 (Fig. 4). Mepolizumab treatment was associated with a significantly greater change from baseline in individual VAS scores compared with placebo at Weeks 49–52 (*P* < 0.001) (Fig. 5). Patients who had undergone ≥ 2 surgeries versus 1 surgery prior to mepolizumab treatment showed less improvement in median change from baseline in loss of sense of smell VAS (Fig. 6).

### SNOT-22 response to treatment

**Fig. 7:**
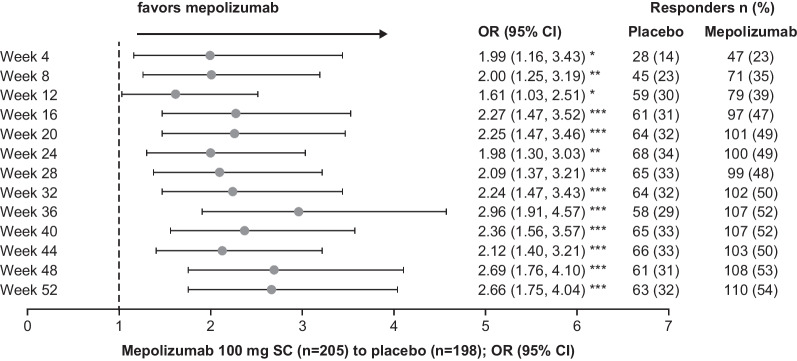
Probability of patients reporting ≥ 28-point improvement from baseline in SNOT-22 total score over time. *P ≤ 0.05; **P ≤ 0.01; ***P ≤ 0.001. OR (mepolizumab vs placebo) of percentage of patients reporting ≥ 28 point improvement from baseline in SNOT-22 total score. OR > 1 indicates greater efficacy of mepolizumab. *CI* Confidence interval, *OR* Odds ratio, *SC* subcutaneous, *SNOT-22* Sino-nasal outcome test-22

**Fig. 8:**
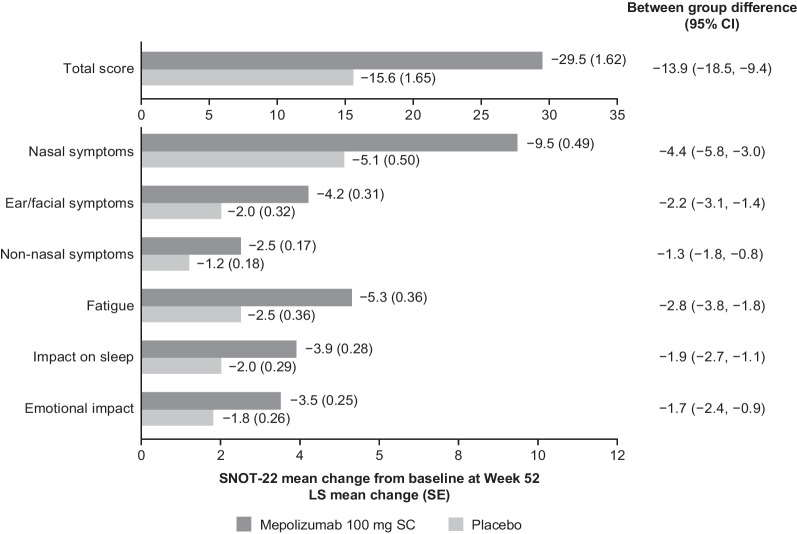
Change from baseline in SNOT-22 total and domain scores at Week 52. Estimates are based on weighting applied to each level of class variable determined from observed proportions. *CI* confidence interval, *LS* Least squares, *SC* Subcutaneous, *SE* Standard error, *SNOT-22* Sino-nasal outcome test-22

A total of 54% and 32% of patients treated with mepolizumab and placebo achieved a ≥ 28-point improvement in SNOT-22 total scores, respectively (Additional file 1: Table S5). Patients receiving mepolizumab had significantly increased odds (OR 1.61–2.96) of achieving a ≥ 28-point improvement in SNOT-22 total score at each 4-weekly time points from Week 4–52 (Fig. 7). There were also significant improvements (*P* < 0.001) in the mean change from baseline in SNOT-22 total and item scores following mepolizumab treatment compared with placebo at Week 52 (Fig. 8).

## Discussion

Using data from SYNAPSE, this analysis evaluated the psychometric properties of the VAS and SNOT-22 in a blinded manner and determined the proportion of responders to mepolizumab using meaningful within-patient change thresholds for these measures, relevant to this population with very severe CRSwNP. Findings indicate that the VAS and SNOT-22 perform well and have acceptable psychometric properties in this very severe CRSwNP population. They also indicate that mepolizumab provided meaningful within-patient improvements in HRQoL versus placebo when added to standard of care. This indicates mepolizumab provides substantial clinical benefits in very severe CRSwNP.

VAS item responses were selected across the range at Weeks 20, 24, and 52. While a higher proportion of responses were clustered at the upper versus lower end of the scale for all symptom VAS scores at baseline, this was expected owing to the very severe CRSwNP SYNAPSE eligibility criteria. No substantial floor or ceiling effects were seen during the treatment period except for a floor effect for loss of sense of smell across all time points. This was likely a result of the CRSwNP and the history of repeat nasal surgery in SYNAPSE patients, which can lead to permanent sense of smell impairment [46, 47]. A floor effect was noted in the facial pain or pressure VAS at Week 52, likely because not all patients experience this symptom [21], and successful mepolizumab treatment will have potentially further decreased this proportion by the end of the study.

All correlations between single VAS items were acceptable, supporting the use of two VAS composite scores (symptoms composite, and nasal symptoms and facial pain composite). Factor structure of the VAS composite scores, as determined by CFA, was acceptable with the CFI and SRMR statistics. Although Chi-square and RMSEA model fit were initially not acceptable, model fit improved following the incorporation of conceptually justifiable residual correlations. Although the residual correlation suggested some redundancy in the VAS items, which can lead to overweighting in composite scoring, these items were still included in the VAS composite scores based on their believed importance to patients. Regarding the Chi-square test, the improvement in model fit may have been due to the model’s tendency to reject the null hypothesis in large samples, such as the 407 patients included in this analysis, even when the hypothesized model shows trivial misfit.

The VAS scores demonstrated acceptable internal consistency and excellent test–retest reliability. The VAS also demonstrated construct validity between corresponding VAS and SNOT-22 items, consistent with a priori hypothesized correlations. Correlations between the VAS and UPSIT, endoscopic NP and PNIF were weaker than a priori hypothesized correlations, which may be indicative of differences between objective or clinician-reported measures and daily patient reports of CRSwNP disease severity. In contrast, correlations between VAS scores and WPAI impairment-based scores and SF-36 PCS exceeded predictions.

The VAS demonstrated acceptable validity based on several known-groups assessments and detected large effect sizes for all VAS scores using all anchors in patients determined to have improved. Psychometric analyses indicated a meaningful within-patient change threshold of − 2.5 points for overall symptoms, nasal discharge, and facial pain VAS and a threshold of − 3.0 points for nasal obstruction, loss of sense of smell, and mucus in throat VAS. This is consistent with previous studies indicating that a change of 2–3 points on a 0–10 VAS is considered a meaningful within-patient change [48, 49], suggesting similar thresholds are appropriate across the spectrum of disease severity as reported elsewhere [50]. Interestingly, while it has been previously assumed that anchor-based mean estimates will be lower than the 95% coefficient of repeatability (underestimating the amount of change needed to be statistically significant at the individual level) [44], our empirical mean estimates were higher. This is likely due to the high reliability estimates observed, resulting in a small degree of measurement error. We also note that the recommended thresholds (guided by CDF and PDF plots in addition to the mean changes) were primarily between the 95% coefficient of repeatability and the anchor-based mean changes.

Psychometric analyses of SYNAPSE confirm the validity of the SNOT-22 total score, as previously reported [14] and support a six-domain structure for reporting SNOT-22 results. Other studies have suggested four [51] or five [52] domains underlying the total score; however, the six-domain structure in this study was indicated by a previous analysis of patients with very severe CRSwNP in a clinical trial setting and thus may be most appropriate for analyzing SYNAPSE data [53]. These psychometric analyses also suggested that a ≥ 28-point improvement (~ 50% change from baseline) in SNOT-22 was an appropriate threshold to determine meaningful within-patient change within this very severe CRSwNP trial population. Different within-patient meaningful change thresholds may be more appropriate in less severe populations or clinical practice; this subject warrants future research. However, a 2010 study by Browne et al., which generated anchor-based values for within-patient meaningful changes for a number of commonly used PROMs suggested little association between baseline severity and within-patient meaningful change values as mathematical coupling can lead to an artificially inflated association between initial value and change score when correlation or regression is used [50]. Such a limitation should be considered when interpreting the results of the present study. In addition, Browne et al., advocated that an MCID should be calculated using a wide range of baseline severities and a single value applied across cohorts; [50] in the case of the SNOT-22 a value of 8.9 points might be appropriate regardless of baseline symptom burden.

The analyses of patient responses to treatment using the VAS and SNOT-22 demonstrated that patients with CRSwNP experienced significant clinical benefits in symptom severity and HRQoL with mepolizumab versus placebo. Furthermore, the effect of mepolizumab on loss of sense of smell measured by the VAS was greatest in patients who had undergone one versus multiple prior surgeries. This may be related to the increased scarring and nerve damage associated with repeat surgeries, limiting the recovery of sense of smell [46, 47]. These results are consistent with results from SYNAPSE, which showed significant improvements in total endoscopic NP score, nasal obstruction VAS and SNOT-22 total score with mepolizumab versus placebo [14].

There were several limitations in the current analyses. Firstly, the psychometric analysis would have ideally used tailored ‘global impression’ anchor measures, specifically designed to evaluate meaningful change thresholds for the VAS and SNOT-22, rather than using the anchoring and triangulation of other outcome measures that were available at the time of this analysis. Use of an arbitrary overall symptoms VAS anchor may have overestimated changes in SNOT-22 score. Given this limitation, care was taken to justify the levels of change on each anchor considered a minimal improvement, where justification was based on clinical insight, published literature and patient input. However, we appreciate that our assumptions on what constitutes a minimal improvement are not guaranteed to precisely capture this level of change, and our chosen definitions may not be universally agreed upon. The suggested exploratory thresholds should therefore be confirmed in future research. Secondly, the same data were used to derive the meaningful within-patient thresholds and to determine the treatment response, which could be considered ‘overfitting’ the data. However, the psychometric analyses used data specific to the SYNAPSE trial in patients with very severe CRSwNP, therefore, the absolute values for respiratory threshold are likely not applicable in a less severe population. Furthermore, blinding the data to establish the thresholds mitigates the potential for overfitting. Thirdly, although the developer-recommended scoring of the SF-36v2 PCS and MCS has been contested, we did not explore alternate scoring options in detail [36, 54]. Finally, the analysis is limited by the SYNAPSE study population of patients with at least one previous nasal surgery [14]. As such, it is important to note that these analyses and their outcomes apply only to the very severe CRSwNP population included in SYNAPSE.

## Conclusions

These psychometric analyses demonstrate that both the VAS and SNOT-22 have acceptable psychometric properties for outcome assessment in patients with very severe CRSwNP. Scores derived from both measures exhibited acceptable internal consistency and construct validity, in addition to test–retest reliability and known-groups validity (assessed only for the VAS). Overall, our analysis demonstrated that patients receiving mepolizumab were more likely to experience improvements in symptom severity and HRQoL versus placebo, when added to standard of care.

## Supplementary Information

**Additional file 1.** Supplementary methods and results.

## Data Availability

Anonymized individual participant data and study documents for the parent study can be requested for further research from www.clinicalstudydatarequest.com.

## Abbreviations

ACQ: Asthma Control Questionnaire
AERD: Aspirin-exacerbated respiratory disease
ANOVA: Analysis of variance
CDF: Cumulative distribution function
CFA: Confirmatory factor analysis
CFI: Comparative fit index
CI: Confidence interval
CRSwNP: Chronic rhinosinusitis with nasal polyps
EPOS: European Position Paper on Rhinosinusitis and Nasal Polyposis
ES: Effect size
HRQoL: Health-related quality of life
ICC: Intra-class correlation
IL: Interleukin
ITT: Intent-to-treat
LS: Least squares
MCID: Minimal clinically important difference
MCS: Mental component summary
N-ERD: Non-steroidal anti-inflammatory drug-exacerbated respiratory disease
NP: Nasal polyps
OR: Odds ratio
PCS: Physical component summary
PDF: Probability density function
PNIF: Peak nasal inspiratory flow
PROMs: Patient-reported outcome measures
RMSEA: Root mean square error of approximation
SC: Subcutaneously
SCS: Systemic corticosteroids
SD: Standard deviation
SE: Standard error
SEM: Standard error of measurement
SF-36: 36-Item Short Form Health Survey
SNOT: Sino-nasal outcome test
SoC: Standard of care
SRMR: Standardized root mean residual
UPSIT: University of Pennsylvania Smell Identification Test
VAS: Visual analogue scale
WPAI: Work productivity and impairment
