# Supplementary material for: Mepolizumab improvements in health-related quality of life and disease symptoms in a patient population with very severe chronic rhinosinusitis with nasal polyps: psychometric and efficacy analyses from the SYNAPSE study
Source: J Patient Rep Outcomes. 2023 Jan 20;7:4. doi: 10.1186/s41687-023-00543-5 (PMC9859976; doi:10.1186/s41687-023-00543-5)
Supplement: Supplementary file 1 — Additional file 1. Supplementary methods and results. [file 41687_2023_543_MOESM1_ESM.docx]

**Supplementary material**

**Mepolizumab improvements in health-related quality of life and disease symptoms in a patient population with very severe chronic rhinosinusitis with nasal polyps: psychometric and efficacy analyses from the SYNAPSE study**

**Contents**

Supplementary Methods

Supplementary Results

**Figure S1.** Study design

**Figure S2.** VAS conceptual framework.

**Figure S3.** Response distributions: nasal obstruction (A–L), nasal discharge (M–T), and overall VAS (U–X).

**Figure S4.** CDF plot of change from baseline to Week 52 in nasal obstruction VAS by A) overall VAS anchor, B) SNOT-22 total score anchor and C) SNOT-22 nasal obstruction anchor.

**Figure S5.** CDF plot of change from baseline to Week 52 in nasal discharge VAS by A) overall VAS anchor, B) SNOT-22 total score anchor and C) SNOT-22 thick nasal discharge anchor.

**Figure S6.** CDF plot of change from baseline to Week 52 in mucus in throat VAS by A) overall VAS anchor, B) SNOT-22 total score anchor and C) SNOT-22 post-nasal discharge anchor.

**Figure S7.** CDF plot of change from baseline to Week 52 in loss of smell VAS by A) overall VAS anchor, B) SNOT-22 total score anchor and C) SNOT-22 loss of taste or smell anchor

**Figure S8.** CDF plot of change from baseline to Week 52 in facial pain VAS by A) overall VAS anchor, B) SNOT-22 total score anchor and C) SNOT-22 facial pain/pressure anchor.

**Figure S9.** CDF plot of change from baseline to Week 52 in overall symptom VAS score by SNOT-22 total score anchor.

**Figure S10.** CDF plot of change from baseline to Week 52 in nasal symptoms composite VAS score by A) overall VAS score anchor and B) SNOT-22 total score; nasal symptoms and facial pain composite VAS score by C) overall VAS score anchor and D) SNOT-22 total score

**Table S1.** Test-retest reliability for symptom VAS and composite VAS scores between Week 20 and Week 24.

**Table S2.** Hypothesized convergent validity associations for each VAS score.

**Table S3.** Proposed anchors.

**Table S4.** Known groups comparison for nasal obstruction VAS scores at baseline and Week 20.

**Table S5.** Proportion of patients with ≥28-point improvement in SNOT-22 total scores at Week 52

**Supplementary Methods**

***SYNAPSE endpoints relevant to this analysis***

- The co-primary endpoints of SYNAPSE were:
  - Change from baseline in total endoscopic NP score at Week 52.
  - Change from baseline in nasal obstruction VAS score during Weeks 49–52.
- Secondary endpoints included:
  - Change from baseline in SNOT-22 total score at Week 52.
  - Change from baseline in loss of sense of smell VAS score at Weeks 49–52.
  - Change from baseline in overall symptoms VAS score at Weeks 49–52.
- Other endpoints included:
  - Change from baseline in nasal discharge, mucus in throat, and facial pain VAS scores at Weeks 49–52.

For full list of endpoints, refer to https://clinicaltrials.gov/ct2/show/NCT03085797.

***Test-retest analysis: stable population sample***

Patients meeting the following definitions between Weeks 20 and 24 were defined as stable:

- Change in endoscopic NP score of 0.
  - Designed to assess reliability of individual symptoms VAS, Overall VAS symptom score, and both composite VAS scores.
- Change in PNIF <20 L/min.
  - Designed to assess reliability of Nasal Obstruction and Loss of Smell VAS scores
- Change in overall symptom VAS score <2.
  - Designed to assess reliability of individual symptoms VAS and both composite VAS scores.
- Change in SNOT-22 total score <8.9.
  - Designed to assess reliability of individual symptoms VAS, Overall VAS symptom score, and both composite VAS scores.
- Change in SNOT-22 nasal obstruction item <2.
  - Designed to assess reliability of Nasal Obstruction VAS score
- Change in SNOT-22 loss of taste or smell item <2.
  - Designed to assess reliability of Loss of Smell VAS score
- Change in SNOT-22 thick nasal discharge item <2.
  - Designed to assess reliability of Nasal Discharge VAS score
- Change in SNOT-22 facial pain/pressure item <2.
  - Designed to assess reliability of Facial Pain VAS score
- Change in SNOT-22 post-nasal discharge item <2.
  - Designed to assess reliability of Mucus in Throat VAS score

***Test-retest reliability***

The formula used to calculate test-retest reliability was:


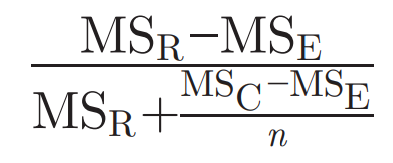


MS_R_, mean square for rows; MS_E_, mean square for error; MS_C_, mean square for columns; 
*n*, number of patients.

***Response to treatment analysis***

Change from baseline in individual and overall symptoms VAS scores, and SNOT-22 total and individual domain scores, and the effect of prior surgeries on loss of sense of smell VAS were assessed at Weeks 49–52 (VAS) or Week 52 (SNOT-22). The analysis of median change from baseline VAS score was performed using quantile regression, with covariates of treatment group, geographic region, baseline score, and log(e) baseline blood eosinophil count. The analysis presented treatment effect as difference in medians to reduce the influence of extreme values on the point estimate of treatment effect. The post hoc analysis of least squares mean change from baseline in SNOT-22 total score was performed using a mixed model repeated measure design with covariates of treatment group, geographic region, baseline score, and log(e) baseline blood eosinophil count, visit plus interaction terms for visit by baseline and visit by treatment group.

**Supplementary Results**

***Confirmatory factor analysis***

For the nasal symptoms composite VAS score model, while the comparative fit index (CFI) (0.986) and standardized root mean square residual (SRMR) (0.017) values were indicative of acceptable fit, Chi-square (24.059) and root mean square error of approximation (RMSEA) (0.167) values were not. Examination of modification indices suggested the inclusion of a residual correlation between Item 1 (nasal obstruction) and Item 4 (loss of smell), which was deemed conceptually justifiable and yielded improved fit (Chi-square *P*=0.079; CFI=0.999; RMSEA=0.073; SRMR=0.007).

For the nasal symptoms and facial pain composite VAS score model, the CFI (0.975) and SRMR (0.020) values were indicative of acceptable fit, whereas Chi-square (58.290) and RMSEA (0.164) values were not. Residual correlations between Item 1 (nasal obstruction) and Item 4 (loss of smell), and between Item 3 (mucus in throat) and Item 5 (facial pain), were suggested by modification indices. Incorporating these residual correlations improved model fit (Chi-square *P*=0.095; CFI=0.998; RMSEA=0.053; SRMR=0.007).

**Supplementary Figures and Tables**

**Figure S1.** Study design


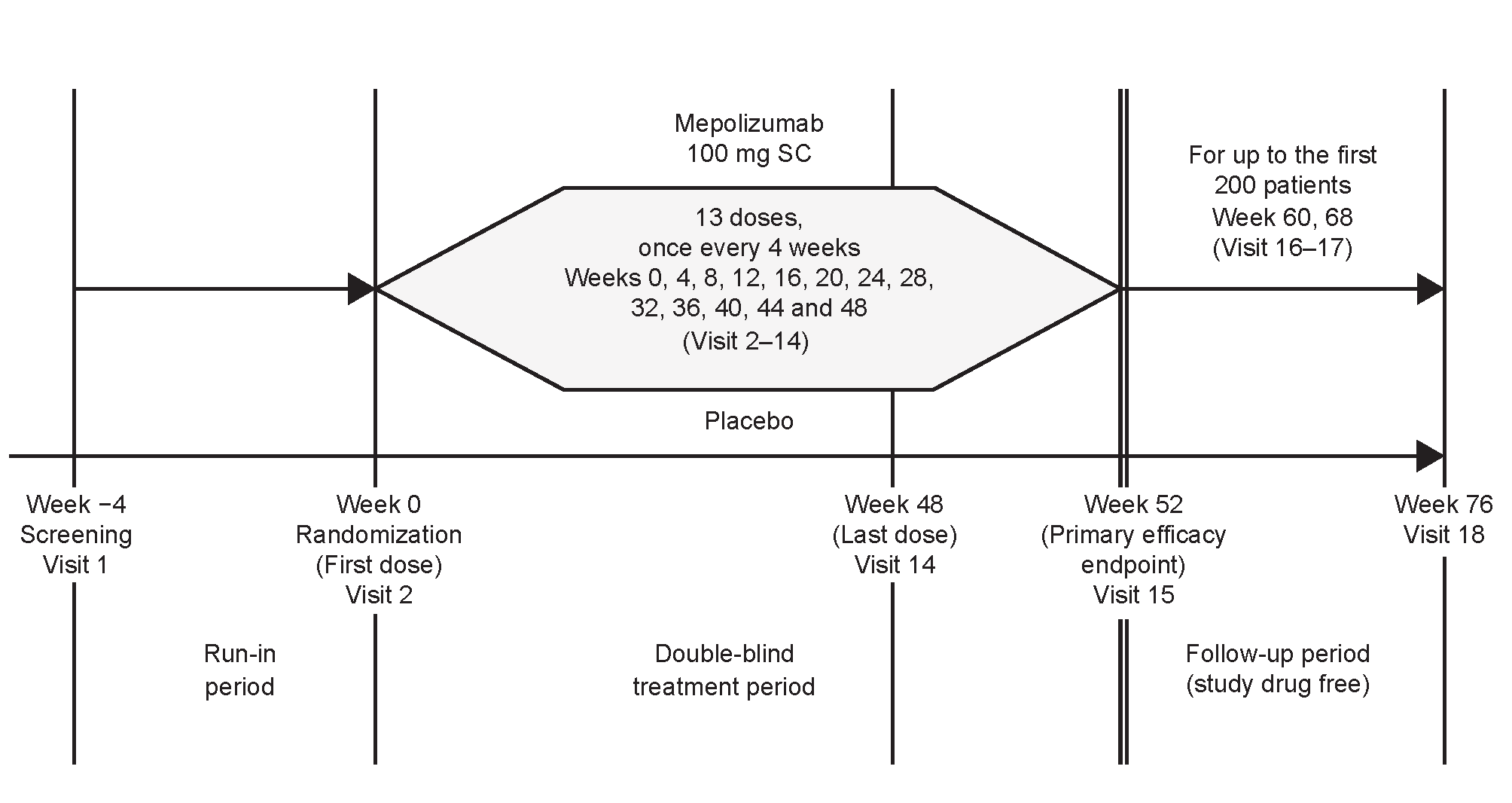
SC, subcutaneous.

**Figure S2.** VAS conceptual framework.


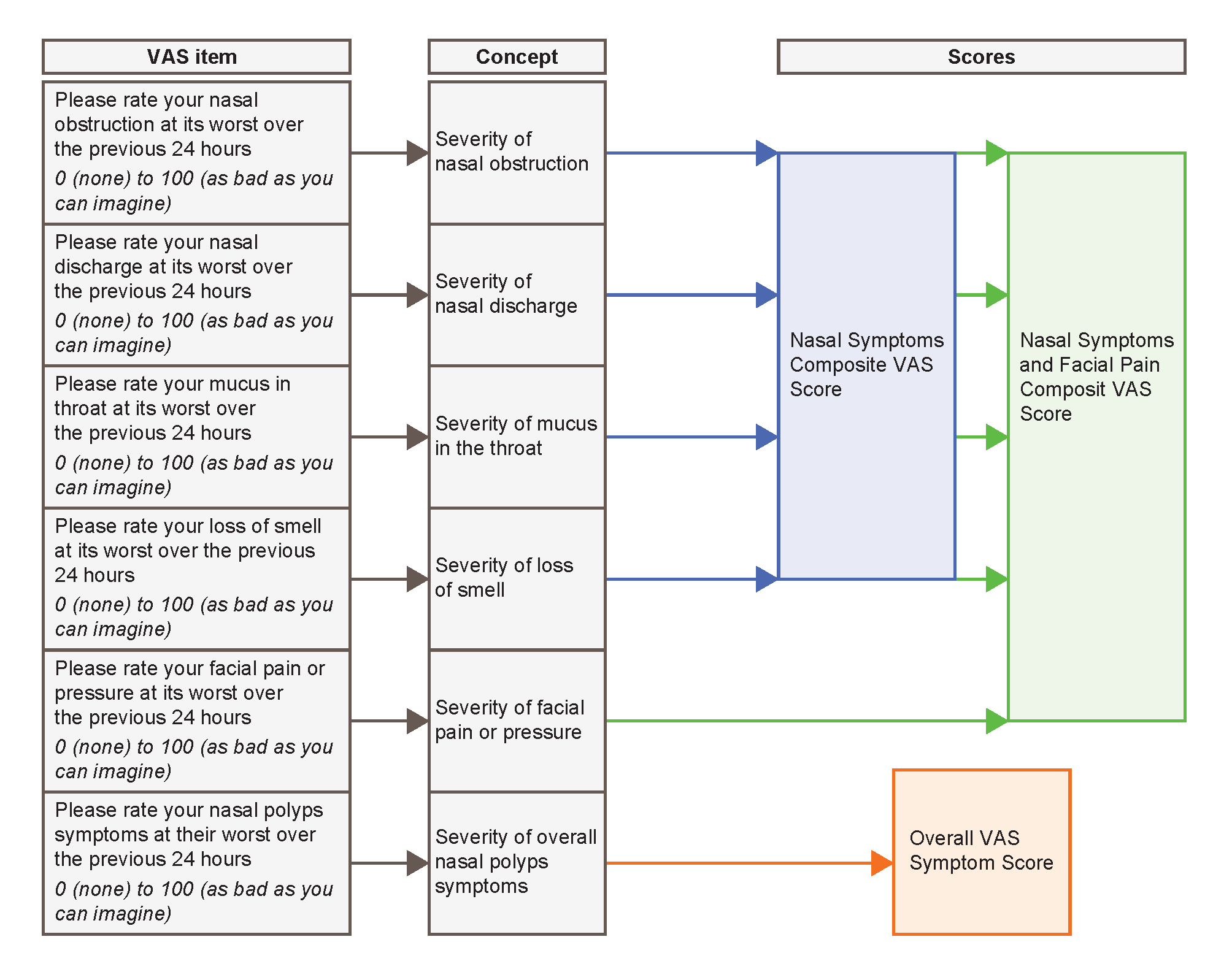


VAS, visual analogue scale.

**Figure S3.** Response distributions: nasal obstruction (A–D), loss of sense of smell (E–H), facial pain (I–L), nasal discharge (M–P), mucus in throat (Q–T) and overall VAS (U–X).

**
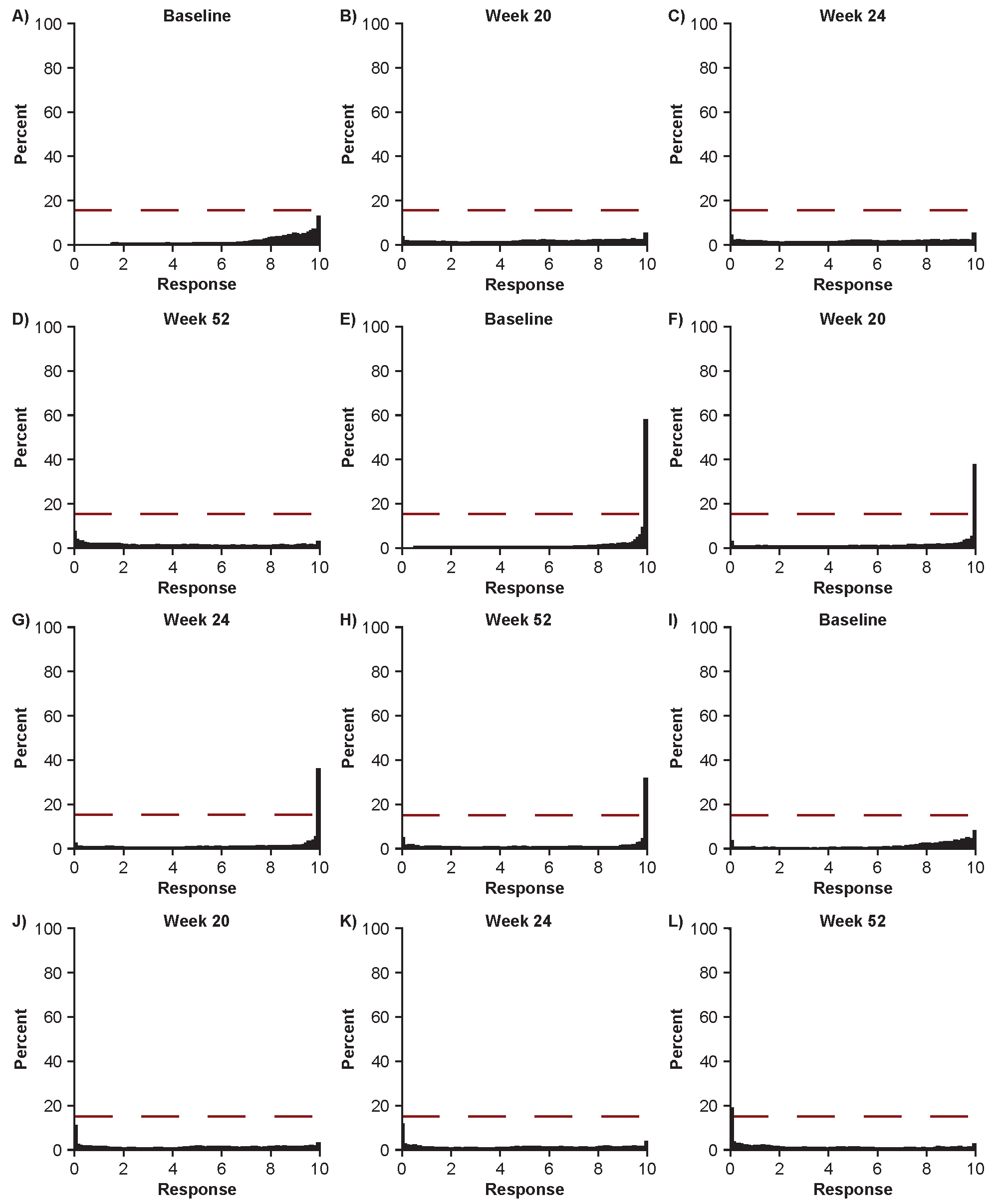
**


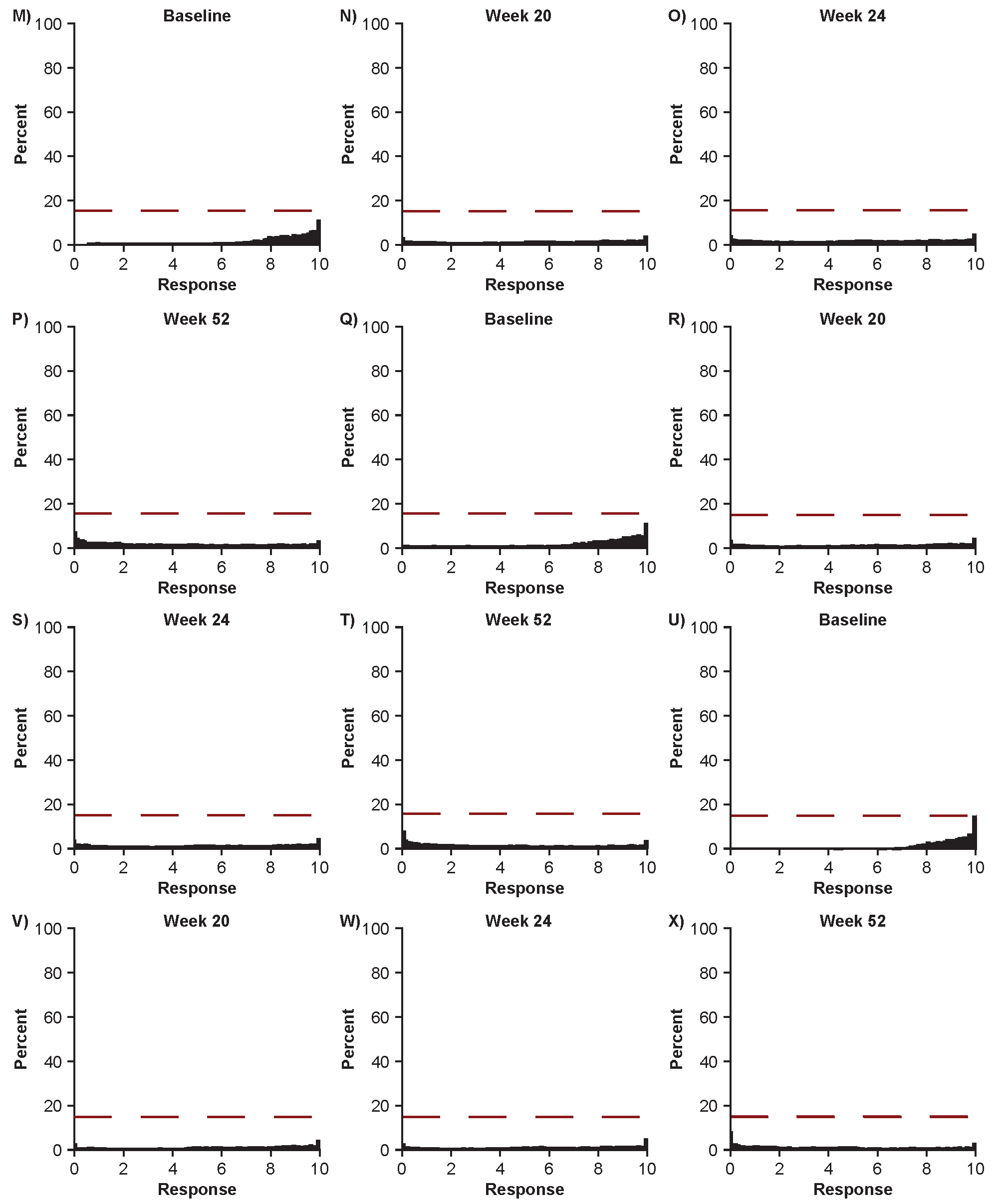
For each VAS, patients rated their symptom on a scale from ‘none’ (0) to ‘as bad as you can imagine’ (100). Results were transformed to a 0–10 scale for reporting (higher scores indicate greater symptom severity). Red dashed line indicates the 15% floor/ceiling threshold.
VAS, visual analogue scale.

**Figure S4.** CDF plot of change from baseline to Week 52 in nasal obstruction VAS by A) overall VAS anchor, B) SNOT-22 total score anchor and C) SNOT-22 nasal obstruction anchor.


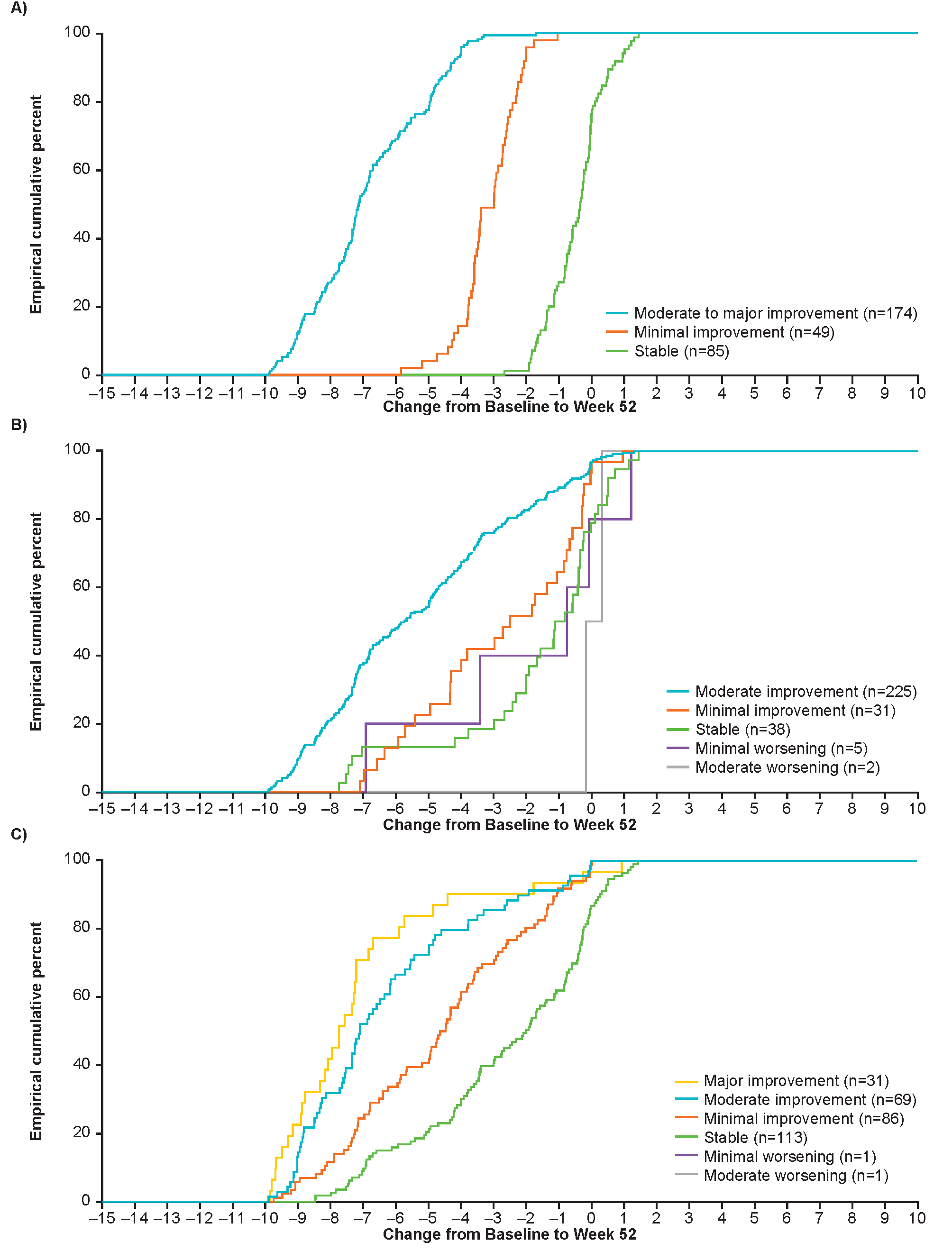


Plotted lines become horizontal when no additional patients within a group have changes from baseline less than the corresponding point on the x-axis.

CDF, cumulative distribution plot; SNOT-22 Sino-nasal Outcome Test-22; VAS, visual analogue scale.**Figure S5.** CDF plot of change from baseline to Week 52 in nasal discharge VAS by A) overall VAS anchor, B) SNOT-22 total score anchor and C) SNOT-22 thick nasal discharge anchor.


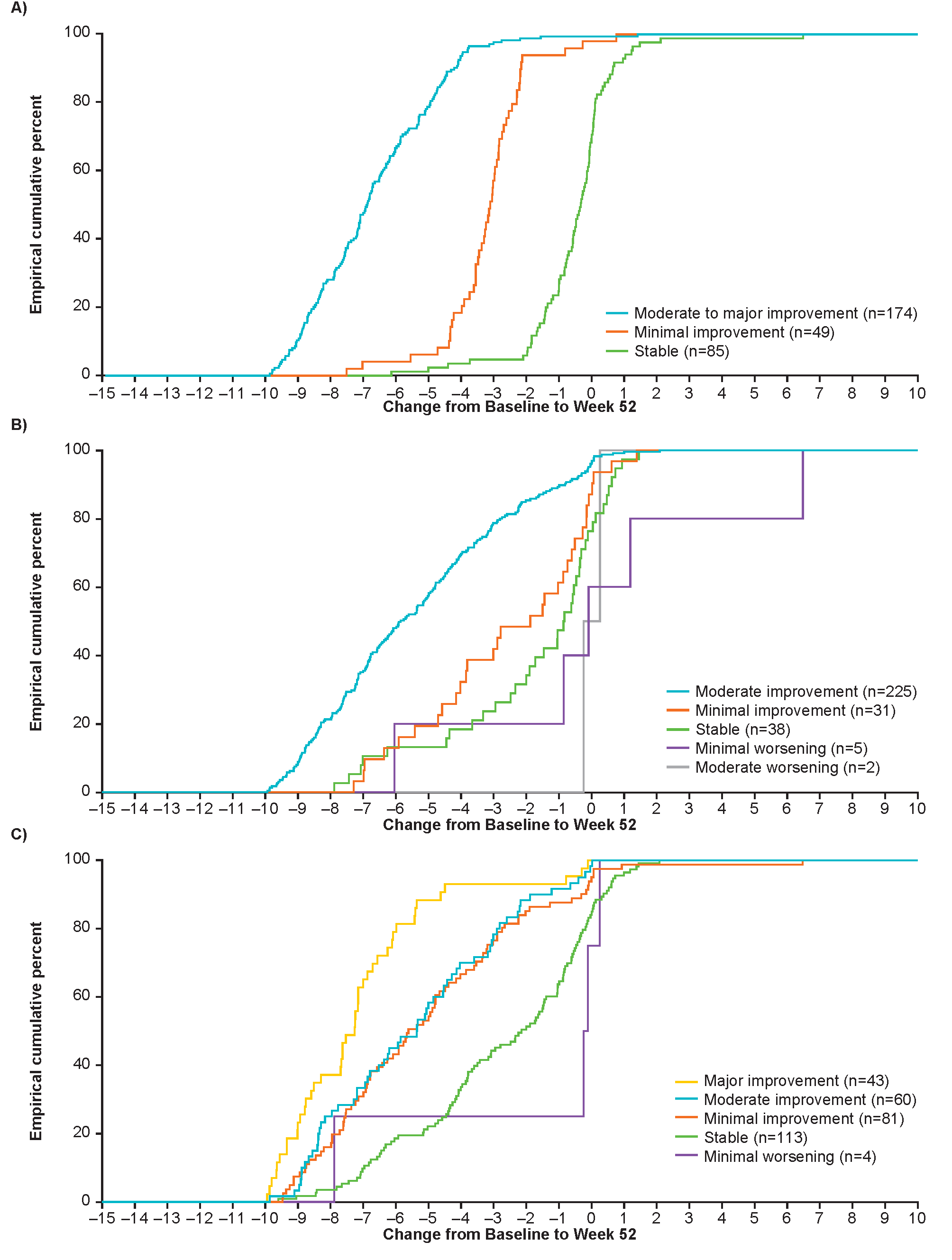


Plotted lines become horizontal when no additional patients within a group have changes from baseline less than the corresponding point on the x-axis.

CDF, cumulative distribution plot; SNOT-22 Sino-nasal Outcome Test-22; VAS, visual analogue scale.

**Figure S6.** CDF plot of change from baseline to Week 52 in mucus in throat VAS by A) overall VAS anchor, B) SNOT-22 total score anchor and C) SNOT-22 post-nasal discharge anchor.


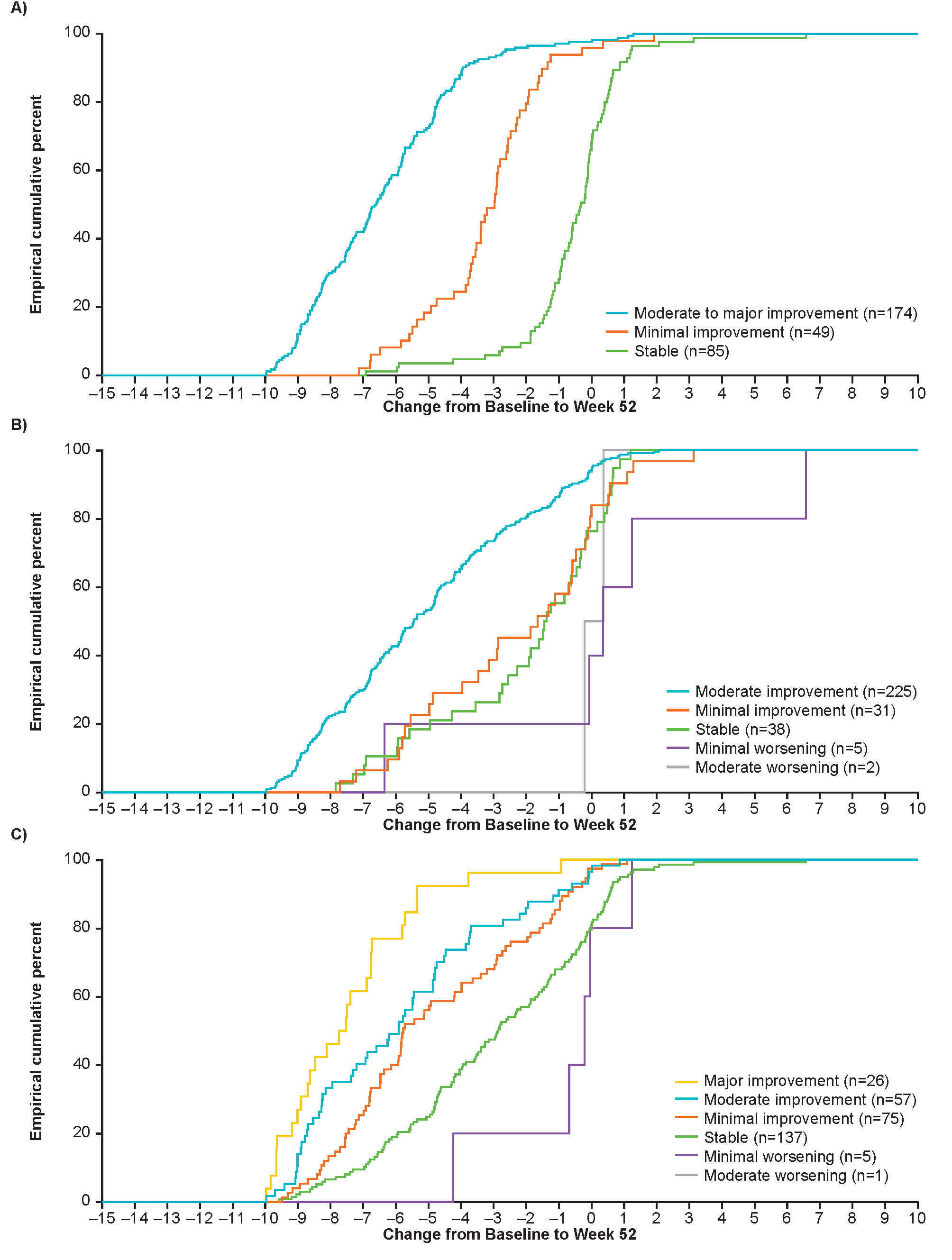


Plotted lines become horizontal when no additional patients within a group have changes from baseline less than the corresponding point on the x-axis.

CDF, cumulative distribution plot; SNOT-22 Sino-nasal Outcome Test-22; VAS, visual analogue scale.

**Figure S7.** CDF plot of change from baseline to Week 52 in loss of smell VAS by A) overall VAS anchor, B) SNOT-22 total score anchor and C) SNOT-22 loss of taste or smell anchor


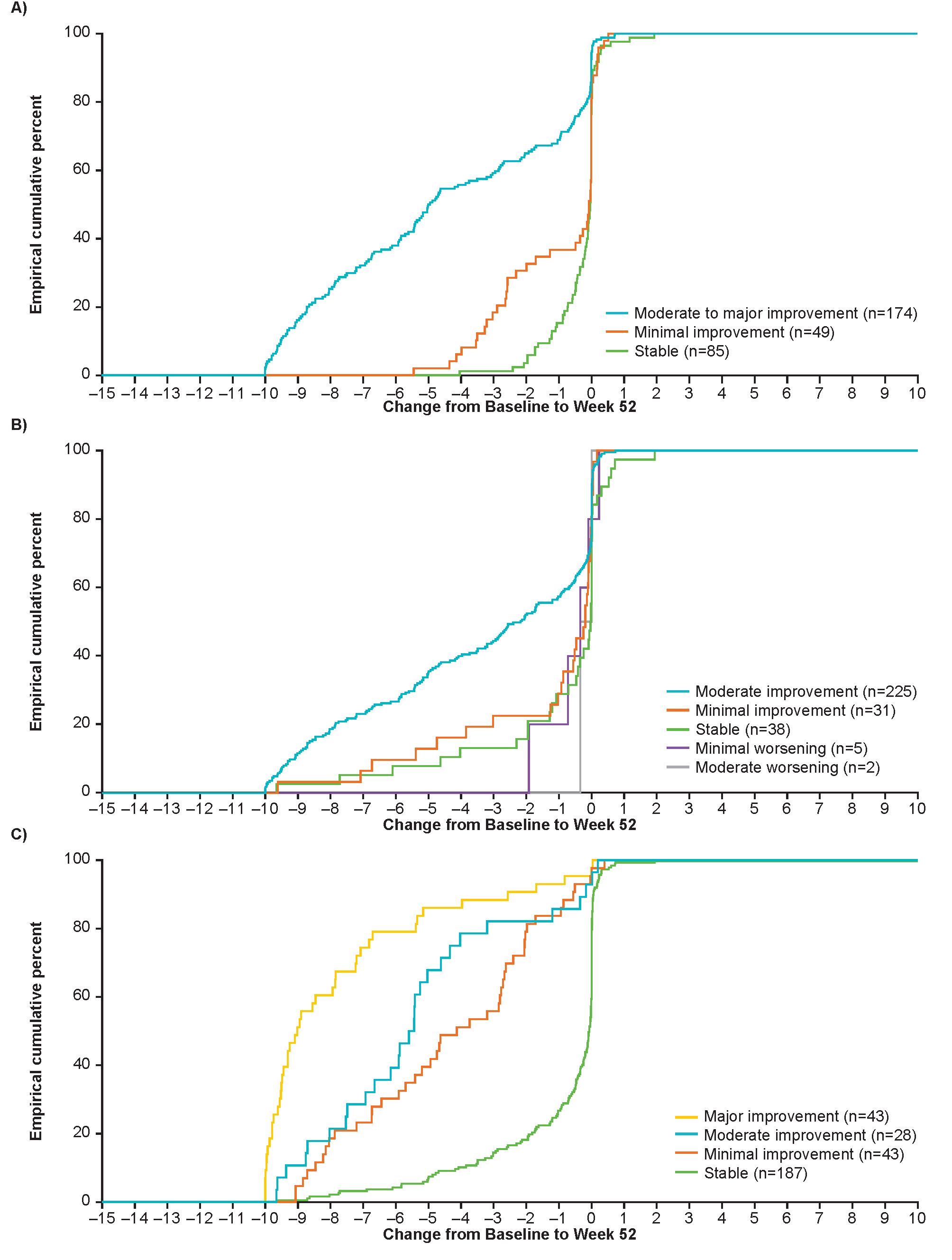


Plotted lines become horizontal when no additional patients within a group have changes from baseline less than the corresponding point on the x-axis.

CDF, cumulative distribution plot; SNOT-22 Sino-nasal Outcome Test-22; VAS, visual analogue scale.

**Figure S8.** CDF plot of change from baseline to Week 52 in facial pain VAS by A) overall VAS anchor, B) SNOT-22 total score anchor and C) SNOT-22 facial pain/pressure anchor.


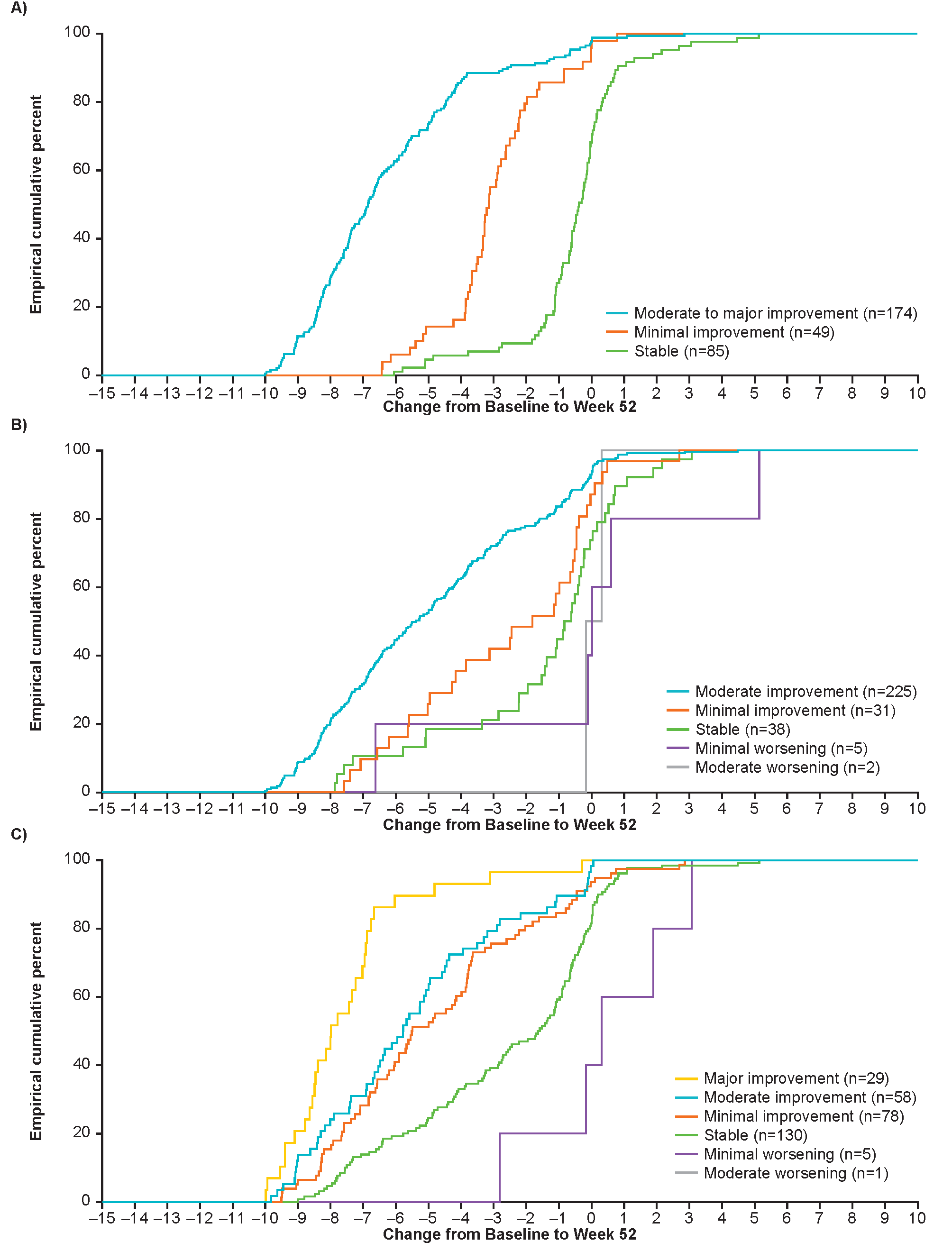


Plotted lines become horizontal when no additional patients within a group have changes from baseline less than the corresponding point on the x-axis.

CDF, cumulative distribution plot; SNOT-22 Sino-nasal Outcome Test-22; VAS, visual analogue scale.

**Figure S9.** CDF plot of change from baseline to Week 52 in overall symptom VAS score by SNOT-22 total score anchor.
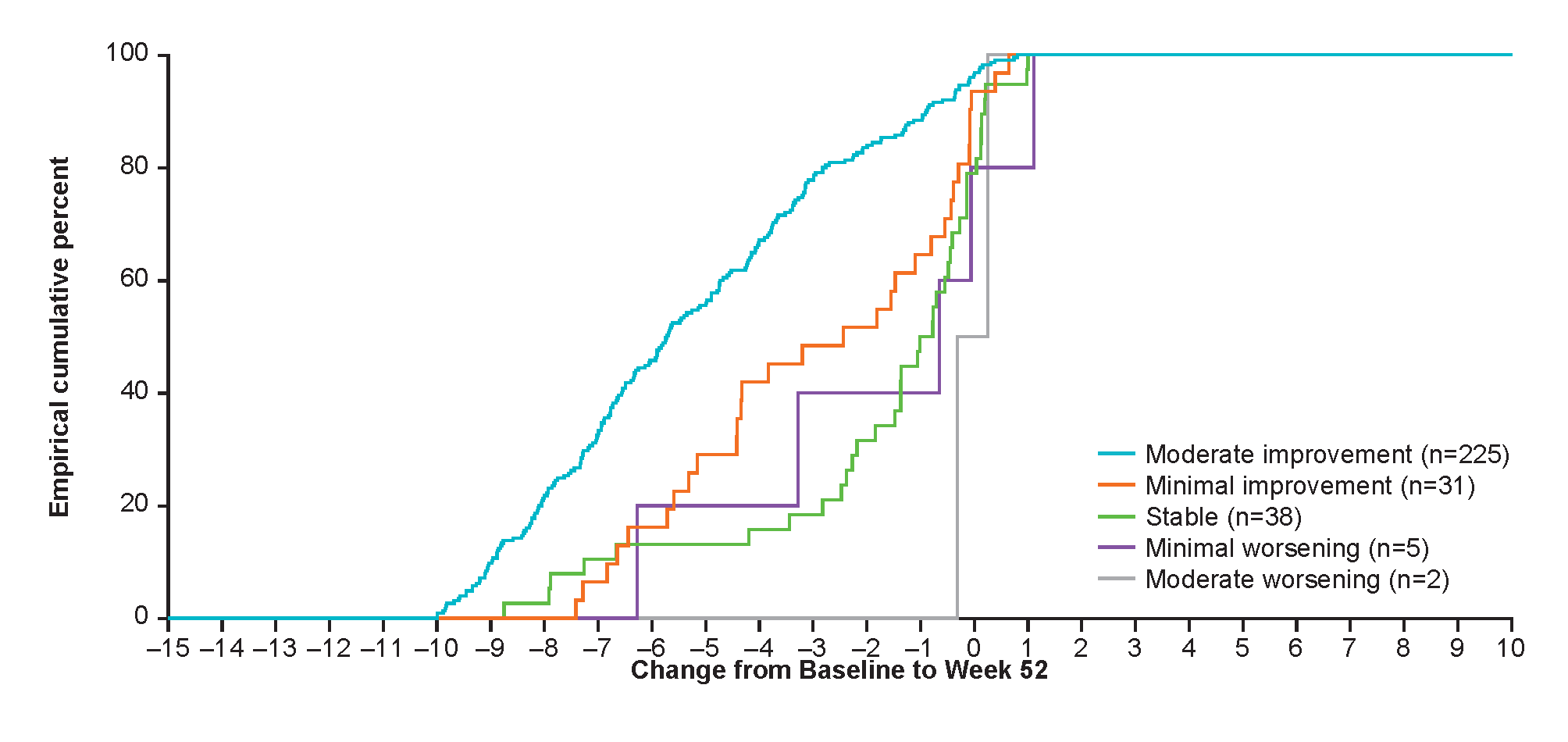


Plotted lines become horizontal when no additional patients within a group have changes from baseline less than the corresponding point on the x-axis.

CDF, cumulative distribution plot; SNOT-22 Sino-nasal Outcome Test-22; VAS, visual analogue scale.

**Figure S10.** CDF plot of change from baseline to Week 52 in nasal symptoms composite VAS score by A) overall VAS score anchor and B) SNOT-22 total score; nasal symptoms and facial pain composite VAS score by C) overall VAS score anchor and D) SNOT-22 total score


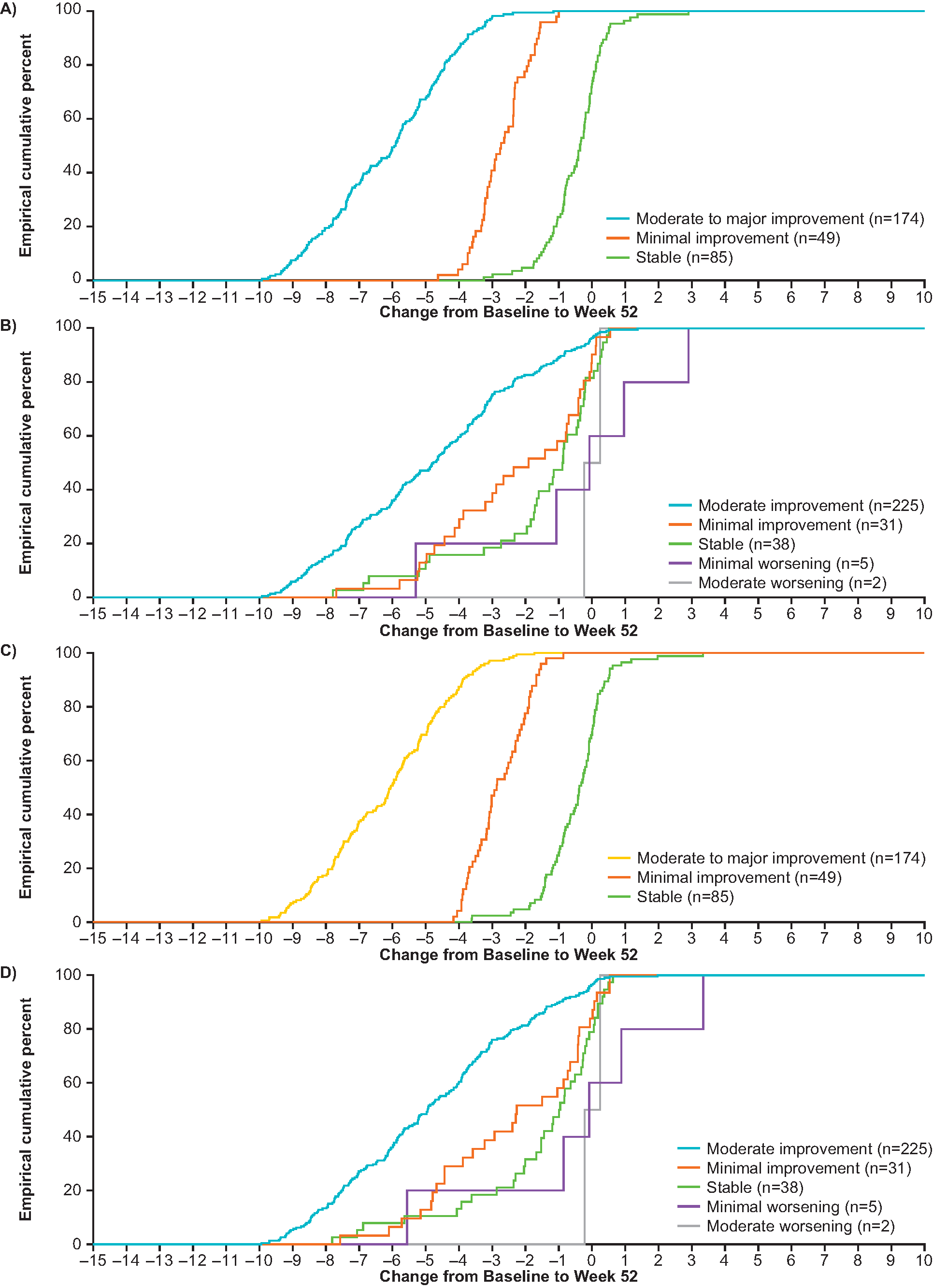


Plotted lines become horizontal when no additional patients within a group have changes from baseline less than the corresponding point on the x-axis.

CDF, cumulative distribution plot; SNOT-22 Sino-nasal Outcome Test-22; VAS, visual analogue scale.

**Table S1.** Test-retest reliability for symptom VAS and composite VAS scores between Week 20 and Week 24.

| Score/Test-retest  Population | N | Intra-Class Correlation  Coefficient (95% CI) |
| --- | --- | --- |
| Nasal Obstruction | | |
| Overall VAS | 358 | 0.987 (0.982, 0.991) |
| Nasal Discharge | | |
| Overall VAS | 358 | 0.988 (0.984, 0.991) |
| Mucus in Throat | | |
| Overall VAS | 358 | 0.989 (0.985, 0.992) |
| Loss of Smell | | |
| Overall VAS | 358 | 0.984 (0.980, 0.988) |
| Facial Pain | | |
| Overall VAS | 358 | 0.991 (0.989, 0.993) |
| Nasal Symptoms Composite VAS Score | | |
| Overall VAS | 358 | 0.989 (0.984, 0.992) |
| Nasal Symptoms and Facial Pain Composite VAS Score | | |
| Overall VAS | 358 | 0.990 (0.986, 0.992) |

Intra-Class Correlation Coefficient is based on a multiple measurement, absolute agreement, two-way mixed effects model.
CI, confidence interval; VAS, visual analogue scale.

**Table S2.** Hypothesized convergent validity associations for each VAS score

|  | UPSIT | Endoscopic NP score | PNIF | WPAI (all scores) | SF-36 (PCS and MCS scores) | SNOT-22 total score | SNOT-22 nasal obstruction | SNOT-22 loss of taste or smell | SNOT-22 thick nasal discharge | SNOT-22 facial pain/ pressure | SNOT-22 post-nasal discharge |
| --- | --- | --- | --- | --- | --- | --- | --- | --- | --- | --- | --- |
| Nasal obstruction | ≥0.50 | ≥0.50 | ≥0.50 | <0.30 | <0.30 | ≥0.30 | ≥0.50 | - | - | - | - |
| Nasal discharge | - | ≥0.30 | - | <0.30 | <0.30 | ≥0.30 | - | - | ≥0.50 | - | - |
| Mucus in throat | - | ≥0.30 | - | <0.30 | <0.30 | ≥0.30 | - | - | - | - | ≥0.50 |
| Loss of sense of smell | ≥0.50 | ≥0.30 | ≥0.50 | <0.30 | <0.30 | ≥0.30 | - | ≥0.50 | - | - | - |
| Facial pain | - | ≥0.30 | - | <0.30 | <0.30 | ≥0.30 | - | - | - | ≥0.50 | - |
| Overall VAS symptoms | ≥0.30 | ≥0.30 | ≥0.30 | <0.30 | <0.30 | ≥0.30 | - | - | - | - | - |
| Nasal symptoms composite VAS score | ≥0.30 | ≥0.30 | ≥0.30 | <0.30 | <0.30 | ≥0.30 | - | - | - | - | - |
| Nasal symptoms and facial pain composite VAS score | ≥0.30 | ≥0.30 | ≥0.30 | <0.30 | <0.30 | ≥0.30 | - | - | - | - | - |

MCS, Mental Component Summary; NP, nasal polyps; PCS Physical Component Summary; PNIF, Peak Nasal Inspiratory Flow; SF-36, 36-item Short Form Health Survey; SNOT-22, Sino-nasal Outcome Test-22; UPSIT, The University of Pennsylvania Smell Identification Test; VAS, visual analogue scale; WPAI, Work Productivity and Activity Impairment questionnaire.

**Table S3.** Proposed anchors.

| **Anchor number** | **Anchor measure** | **Definition** | **Justification** |
| --- | --- | --- | --- |
| 1. | Endoscopic NP score | • Extreme improvement: ≤-4  • Major improvement: -3  • Moderate improvement: -2  • Minimal improvement: -1  • Stable: 0  • Minimal worsening: +1  • Moderate worsening: +2  • Major worsening: ≥3 | Minimal improvement aligned with responder definition applied in Study 205687 |
| 2. | PNIF | • Moderate improvement: ≥40 L/min  • Minimal improvement: 40 > change score ≥20 L/min  • Stable: -20 < change score <20  • Minimal worsening: -40 < change score ≤-20 L/min  • Moderate worsening: ≤-40 L/min | Based on published threshold for PNIF [[30](#_ENREF_30)] |
| 3. | Overall VAS symptom score | • Moderate to major improvement: ≤-4  • Minimal improvement: -2 ≥ change score >-4  • Stable: -2 < change score <2  • Minimal worsening: 2 ≤ change score <4  • Moderate worsening:  4 ≤ change score <6  • Major worsening: ≥6 | Cognitive debriefing of the VAS in patients with NP suggested 20–39% reductions could be meaningful (7/21 participants). This reduction from a starting point of 7 equates to 2–3 points. |
| 4. | SNOT-22 total score | • Moderate improvement: ≤-17.8  • Minimal improvement: -8.9 ≥ change score >-17.8  • Stable: -8.9 < change score <8.9  • Minimal worsening: 8.9 < change score ≤17.8  • Moderate worsening: ≥17.8 | Minimal improvement aligned with responder definition applied in Study 205687. |
| 5. | SNOT-22 ‘nasal obstruction’ | • Major improvement:  ≤-4  • Moderate improvement: -3  • Minimal improvement: -2  • Stable: -2 < change score <2  • Minimal worsening: +2  • Moderate worsening: +3  • Major worsening: ≥4 | Cognitive debriefing of the item in patients with NP who were currently severe suggested 2–3 point reductions are meaningful (4/6 severe participants). |
| 6. | SNOT-22 ‘loss of taste or smell’ | • Major improvement:  ≤-4  • Moderate improvement: -3  • Minimal improvement: -2  • Stable: -2 < change score <2  • Minimal worsening: +2  • Moderate worsening: +3  • Major worsening: ≥4 | Cognitive debriefing of the item in patients with NP who were currently severe suggested 2–3 point reductions are meaningful (4/8 severe participants). |
| 7. | SNOT-22 ‘thick nasal discharge’ | • Major improvement:  ≤-4  • Moderate improvement: -3  • Minimal improvement: -2  • Stable: -2 < change score <2  • Minimal worsening: +2  • Moderate worsening: +3  • Major worsening: ≥4 | Cognitive debriefing of the item in patients with NP who were currently severe suggested 2–3 point reductions are meaningful (3/5 severe participants). |
| 8. | SNOT-22 ‘facial pain/pressure’ | • Major improvement: ≤-4  • Moderate improvement: -3  • Minimal improvement: -2  • Stable: -2 < change score <2  • Minimal worsening: +2  • Moderate worsening: +3  • Major worsening: ≥4 | Cognitive debriefing of the item in patients with NP who were currently severe suggested 2–3 point reductions are meaningful (7/8 severe participants). |
| 9. | SNOT-22 ‘post-nasal discharge’ | • Major improvement: ≤-4  • Moderate improvement: -3  • Minimal improvement: -2  • Stable: -2 < change score <2  • Minimal worsening: +2  • Moderate worsening: +3  • Major worsening: ≥4 | Cognitive debriefing of the item in patients with NP who were currently severe suggested 2–3 point reductions are meaningful (5/8 severe participants). |

All anchors were not designed for use with all scores. Anchors 4–9 did not inform score interpretation analyses for the SNOT-22 Total score or Overall VAS symptom scores. Anchors 5–9 were specifically designed to inform analyses of each of the five individual symptom VAS scores and were of lesser importance in analyses for composite VAS scores and SNOT-22 Total Score.
NP, nasal polyps; PNIF, Peak Nasal Inspiratory Flow; SNOT-22, Sino-nasal Outcome Test-22; VAS, visual analogue scale.

**Table S4.** Known groups comparison for nasal obstruction VAS scores at baseline and Week 20.

| **Week** | **Known-groups** | **N** | **Mean (SD)** | **Effect size*** | ***P*-value^†^** |
| --- | --- | --- | --- | --- | --- |
| **Baseline** | Participants with comorbid asthma  Group 1: No concurrent asthma  Group 2: Concurrent asthma | 118  289 | 8.9 (0.87)  9.0 (0.81) | -0.20 | 0.0669 |
|  | Blood eosinophil count categories  Group 1: ≤0.30  Group 2: >0.30 | 135  272 | 8.9 (0.77)  9.0 (0.86) | -0.13 | 0.2352 |
|  | Blood eosinophil count categories  Group 1: ≤0.15  Group 2: >0.15 | 40  367 | 9.0 (0.74)  9.0 (0.84) | 0.08 | 0.6210 |
|  | AERD  Group 1: No current AERD  Group 2: Current AERD | 299  108 | 8.9 (0.80)  9.1 (0.89) | -0.24 | 0.0369 |
|  | ACQ-5  Group 1: Well-controlled asthma  Group 2: Partially controlled asthma  Group 3: Inadequately controlled asthma | 46  48  188 | 9.0 (0.75)  8.9 (0.81)  9.1 (0.83) | 0.13  -0.11 | 0.3433 |
|  | Number of prior surgeries  Group 1: 1  Group 2: 2  Group 3: >2 | 189  94  124 | 8.9 (0.80)  9.0 (0.84)  9.0 (0.87) | -0.09  -0.15 | 0.4304 |
| **Week 20** | Participants with comorbid asthma  Group 1: No concurrent asthma  Group 2: Concurrent asthma | 111  275 | 5.9 (2.86)  6.1 (2.96) | -0.08 | 0.4768 |
|  | AERD  Group 1: No current AERD  Group 2: Current AERD | 285  101 | 6.1 (2.86)  6.0 (3.13) | 0.01 | 0.9072 |
|  | ACQ-5  Group 1: Well-controlled asthma  Group 2: Partially controlled asthma  Group 3: Inadequately controlled asthma | 106  73  87 | 5.1 (3.06)  6.0 (2.79)  7.4 (2.48) | -0.33  -0.83 | <0.0001 |
|  | Number of prior surgeries  Group 1: 1  Group 2: 2  Group 3: >2 | 181  90  115 | 5.6 (2.90)  6.6 (2.81)  6.3 (2.99) | -0.34  -0.22 | 0.0208 |

*Between-group standardized effect size calculated as Cohen’s d; †*P*-values are derived using a one-way ANOVA F-test for mean differences between groups. ACQ, Asthma Control Questionnaire; AERD, aspirin-exacerbated respiratory disease; ANOVA, analysis of variance; SD, standard deviation.

**Table S5.** Proportion of patients with ≥28-point improvement in SNOT-22 total scores at Week 52

|  | Placebo (N=201) | Mepolizumab 100 mg SC (N=206) |
| --- | --- | --- |
| n | 198 | 205 |
| Met ≥28-point improvement, n (%) | 63 (32) | 110 (54) |
| Not met ≥28-point improvement, n (%) | 135 (65) | 95 (46) |
| <28-point improvement, n (%) | 56 (28) | 48 (23) |
| No change/worsening | 15 (8) | 9 (4) |
| Nasal surgery prior to visit | 45 (23) | 18 (9) |
| Withdrawn from study prior to visit | 13 (7) | 16 (8) |
| Missing visit data | 6 (3) | 4 (2) |

Analysis was performed using a logistic regression model with covariates of treatment group, geographic region, baseline score and log(e) baseline blood eosinophil count.

SC, subcutaneous; SNOT-22, Sino-nasal Outcome Test-22.
